# FDX1 overexpression inhibits the growth and metastasis of clear cell renal cell carcinoma by upregulating FMR1 expression

**DOI:** 10.1038/s41420-025-02380-5

**Published:** 2025-03-21

**Authors:** Wuping Yang, Cunjin Wu, Chaochao Jiang, Taile Jing, Minghao Lu, Dan Xia, Ding Peng

**Affiliations:** 1 Department of Urology, The First Affiliated Hospital, Zhejiang University School of Medicine, Hangzhou, PR China; 2 Department of Urology, Changxing Hospital of Traditional Chinese Medicine, Changxing, PR China

**Keywords:** Renal cell carcinoma, Tumour-suppressor proteins

## Abstract

Kidney cancer has caused more than 150,000 deaths in 185 countries around the world and is a serious threat to human life. Clear cell renal cell carcinoma (ccRCC) is the most common type of kidney cancer. FDX1, a crucial gene for regulating copper death, plays an important role in tumors. However, its specific role in ccRCC remains unclear. In this study, by analysing data from the TCGA-KIRC and GEO databases and validation in clinical samples from our center, the expression characteristics of FDX1 and its relationship with tumor clinicopathological features and patient prognosis were clarified; the effects of FDX1 overexpression on ccRCC cell proliferation, apoptosis, migration, and invasion were determined via cell phenotype experiments and mouse orthotopic renal tumor growth models; and the downstream regulatory mechanism of FDX1 was determined via TMT proteomic sequencing, Co-IP assays, and RNA-sequencing detection. Our results confirmed that FDX1 was significantly underexpressed in ccRCC and that reduced FDX1 expression was associated with adverse clinicopathologic features and poor prognosis. FDX1 overexpression markedly inhibited the proliferation, migration, and invasion of ccRCC cells and promoted cell apoptosis in vitro. Mechanistically, FDX1 bound to the FMR1 protein and upregulated its expression, subsequently restraining Bcl-2 and N-cadherin expression and enhancing ALCAM, Cleaved Caspase-3, and E-cadherin expression. In mouse models, FDX1 overexpression significantly suppressed the growth and metastasis of renal tumors, but this inhibitory effect was markedly reversed after FMR1 expression was knocked down. Thus, our results confirmed that FDX1 expression is significantly reduced in ccRCC and serves as a prognostic marker for ccRCC patients and that its overexpression suppresses the growth and metastasis ability of ccRCC by promoting the expression of FRM1.

## Introduction

As one of the three most common malignant tumors in the urinary system, kidney cancer reported over 430,000 new cases and caused over 150,000 deaths in 185 countries worldwide in 2022, ranking 14th and 16th among 36 types of cancer, respectively [1]. Thus, kidney cancer threatens the survival of human beings throughout society. Renal cell carcinoma (RCC) is the most common type of kidney cancer, accounting for approximately 85% of all cases, with clear cell RCC (ccRCC) accounting for 75% or more of RCCs [2]. Currently, nearly 1/3 of ccRCC patients have already experienced distant metastasis at the time of diagnosis and drug therapy is the main treatment option for most patients during this period [3]. In the past decade, many targeted drugs and immune checkpoint inhibitors have been applied clinically and have improved patient survival to some extent. However, targeted therapy is prone to developing drug resistance, whereas immunotherapy is only effective for certain specific populations [3–5]. Thus, there is a constant need to find new targets for advanced ccRCC treatment to provide patients with more treatment options.

The unique cellular mechanism by which copper directly binds to the fatty acid-acylated component of the tricarboxylic acid cycle, resulting in toxic protein stress and cell death, is known as cuproptosis [6]. The small iron–sulfur (Fe–S) protein encoded by the ferritin 1 (*FDX1*) gene has the characteristics of low redox potential, low molecular weight, and the presence of at least one Fe–S cluster [7]. Many recent articles have shown that FDX1 is an important gene that regulates copper death and plays a vital role in tumors [8–12]. There are also many studies on FDX1 in ccRCC. For example, when TCGA-KIRC data were analysed, the FDX1 expression level in ccRCC was significantly lower than that in adjacent normal (AN) tissue, and lower FDX1 expression was closely associated with adverse pathological features and shorter overall survival (OS) of patients [6]. According to data from multiple public databases, bioinformatics analysis results revealed that FDX1 was abnormally expressed in ccRCC and confirmed that FDX1 could serve as a direct target to effectively promote the killing effect of copper ionophores [13]. Although these studies preliminarily suggested that FDX1, a copper death related gene, may be dysregulated in ccRCC and play a certain role in this process, most of these results were obtained through analysis of public databases, which lack more clinical sample validation and deeper mechanistic exploration.

In the present study, we aimed to further clarify the expression pattern, clinical significance and biological functions of FDX1 in ccRCC, and explored its downstream regulatory mechanisms in greater depth. Our preliminary experimental results verified that FDX1 expression was significantly decreased in ccRCC and that decreased FDX1 expression was associated with adverse clinicopathological characteristics of tumors and poor patient prognosis. Then, cell phenotype experiments and mouse orthotopic renal tumor growth models were used to clarify the biological function of FDX1 in ccRCC. Moreover, TMT proteomic sequencing, Co-IP assays and RNA-seq were utilised to explore the regulatory mechanism of FDX1 in ccRCC. Our findings were expected to identify a new mechanism by which FDX1 inhibits the progression of ccRCC and provide new targets for the treatment of ccRCC.

## Results

### FDX1 expression is decreased in ccRCC and its reduced expression is associated with poor prognosis based on public databases

**Fig. 1:**
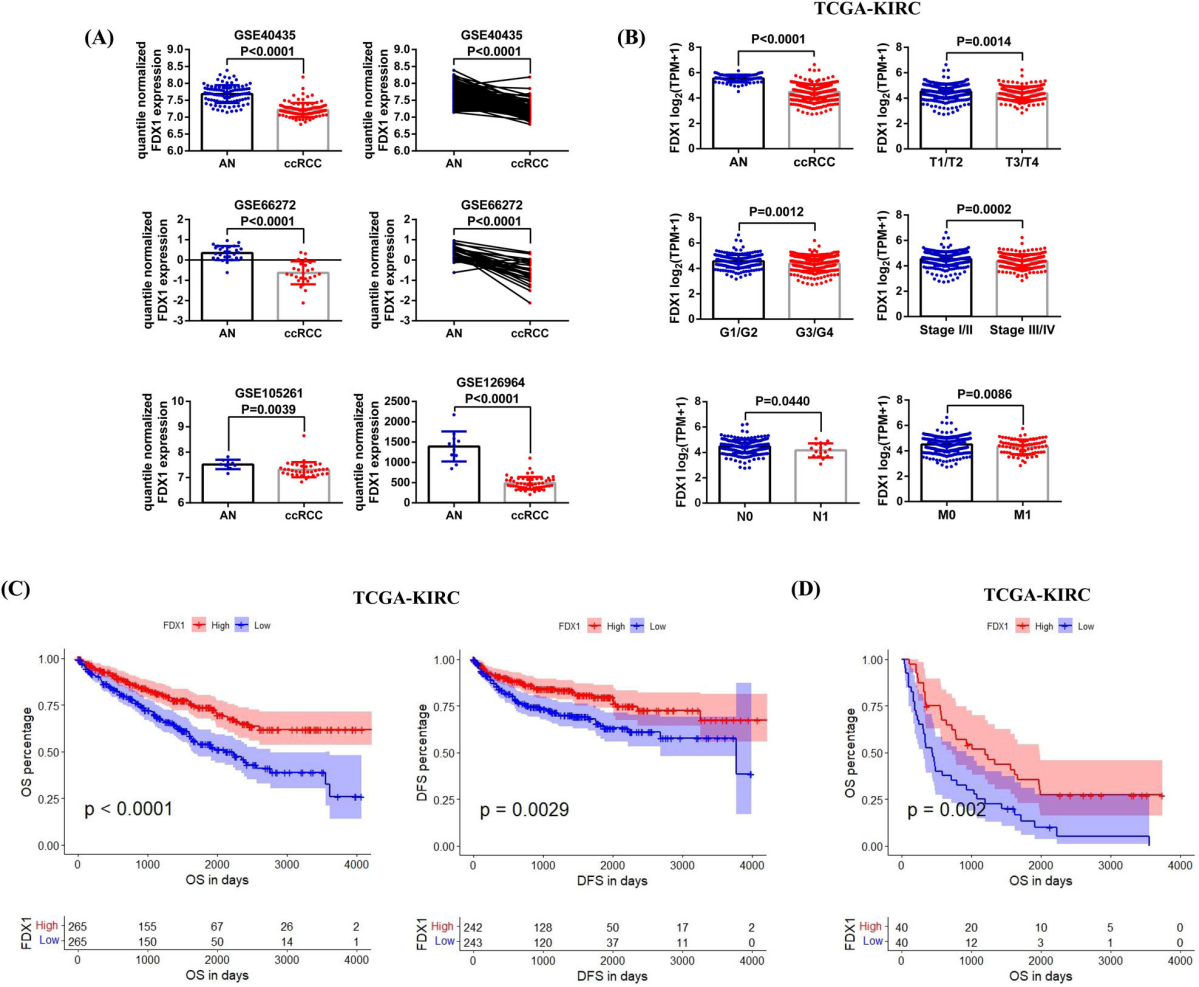
The expression characteristics and clinical significance of FDX1 in ccRCC according to public databases. **A** Comparison of FDX1 expression between ccRCC and AN tissues based on the data of 4 GSE datasets (GSE40435, GSE66272, GSE105261 and GSE126964). **B** Comparison of FDX1 expression between ccRCC and AN tissues and its expression differences in tumors with different pathological features based on TCGA-KIRC data. **C** Analysis of the relationships between FDX1 expression and the OS and DFS of patient based on TCGA-KIC data. **D** The relationship between FDX1 expression and the OS of patient with distant metastasis.

First, we collected the expression data of FDX1 in ccRCC and adjacent normal (AN) tissues from 4 GSE datasets (GSE40435, GSE66272, GSE105261, and GSE126964), and conducted analyses and comparisons. The results revealed that FDX1 mRNA expression was significantly lower in ccRCC than in AN tissues (Fig. 1A). Next, we compared FDX1 expression in ccRCC and AN tissues on the basis of TCGA-KIRC data. The results also indicated that FDX1 expression was markedly decreased in ccRCC and that reduced FDX1 expression was associated with higher tumor stage, grade, lymph node invasion, and distant metastasis (T3/T4, G3/G4, Stage III/IV, N1, and M1) (Fig. 1B). In addition, by combining the survival information of patients for prognostic analysis, the results demonstrated that, compared with patients in the FDX1 high-expression group, patients in the FDX1 low-expression group had shortened Overall Survival (OS) and Disease Free Survival (DFS) (Fig. 1C). In addition, we divided these patients with distant metastasis into high- and low-FDX1 expression groups on the basis of the median expression of FDX1 for prognostic analysis. The results also confirmed that patients in the FDX1 low-expression group had shorter OS than those in the FDX1 high-expression group did (Fig. 1D).

### Validation of the expression and clinical significance of FDX1 in clinical ccRCC samples

**Fig. 2:**
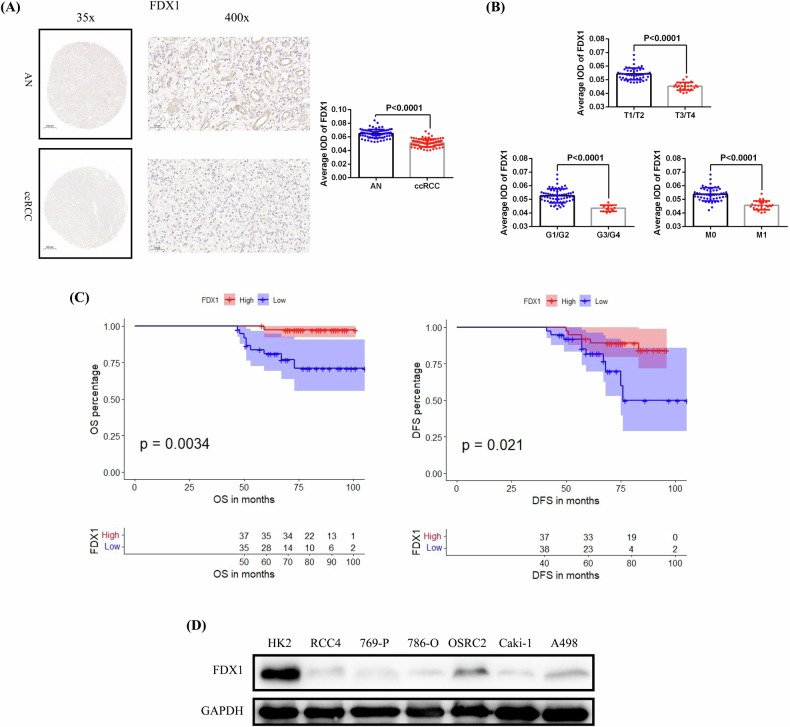
Validation of the expression characteristics and clinical significance of FDX1 in ccRCC. **A** The expression of FDX1 in 75 pairs of ccRCC and AN tissues detected by immunohistochemistry. **B** Comparison of the differences of FDX1 expression in tumors with different pathological features. **C** Analysis of the relationships between FDX1 expression and the OS and DFS of patients. **D** Detection of FDX1 protein level in ccRCC cell lines by western blot.

To further clarify the expression characteristics of FDX1 in ccRCC, we used immunohistochemistry (IHC) to detect the protein expression of FDX1 in paraffin sections of 75 pairs of ccRCC and AN tissues from our center and conducted a comparative analysis. Our results confirmed that FDX1 protein expression was significantly downregulated in ccRCC tissues (Fig. 2A), and its expression was lower in tumors with higher stages, grades, and distant metastases (T3/T4, G3/G4, and M1) (Fig. 2B). The results of the prognostic analysis based on the survival information of these patients determined that the OS and DFS of patients in the FDX1 low-expression group were also significantly shorter than those in the FDX1 high-expression group (Fig. 2C). In addition, we used western blot to detect the protein expression of FDX1 in 6 ccRCC lines and compared it with its expression in the HK2 cell line. Our results indicated that, FDX1 protein expression was markedly lower in the RCC4, 769-P, 786-O, OSRC2, Caki-1, and A498 cell lines than in the HK2 cells, (Fig. 2D). Therefore, our above results confirmed the low-expression pattern of FDX1 in ccRCC and suggest that FDX1 could serve as a prognostic marker for ccRCC patients.

### The effect of FDX1 overexpression on the phenotype of ccRCC cells in vitro

**Fig. 3:**
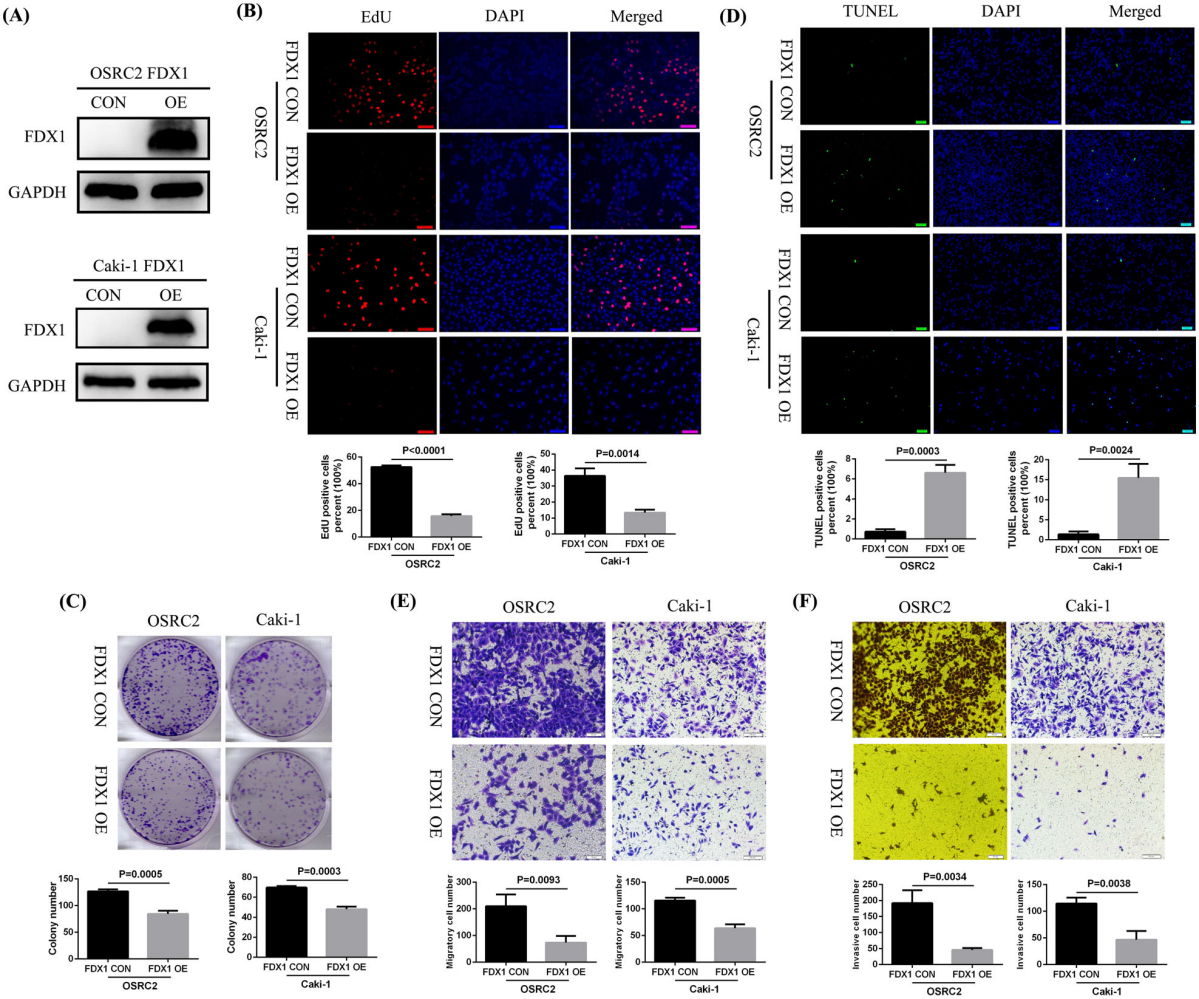
The effects of FDX1 overexpression on the phenotype of ccRCC cells in vitro. **A** Successful overexpression of FDX1 in ccRCC cells. **B**–**F** The effect of FDX1 overexpression on cell proliferation (EdU), cell growth (colony formation assay), cell apoptosis (TUNEL), cell migration, and cell invasion, respectively.

To clarify the biological function of FDX1 in ccRCC, we successfully overexpressed FDX1 protein in the OSRC2 and Caki-1 cell lines (Fig. 3A). The effects of FDX1 overexpression on ccRCC cell proliferation, apoptosis, migration, and invasion were detected via the immunofluorescence EdU method, plate cloning experiment, TUNEL immunofluorescence, cell migration experiment, and cell invasion experiment, respectively. Our experimental results confirmed that FDX1 overexpression significantly suppressed cell proliferation (Fig. 3B, C), promoted cell apoptosis (Fig. 3D), and restrained cell migration and invasion ability (Fig. 3E, F).

### FDX1 binds to the FMR1 protein and promotes its expression

**Fig. 4:**
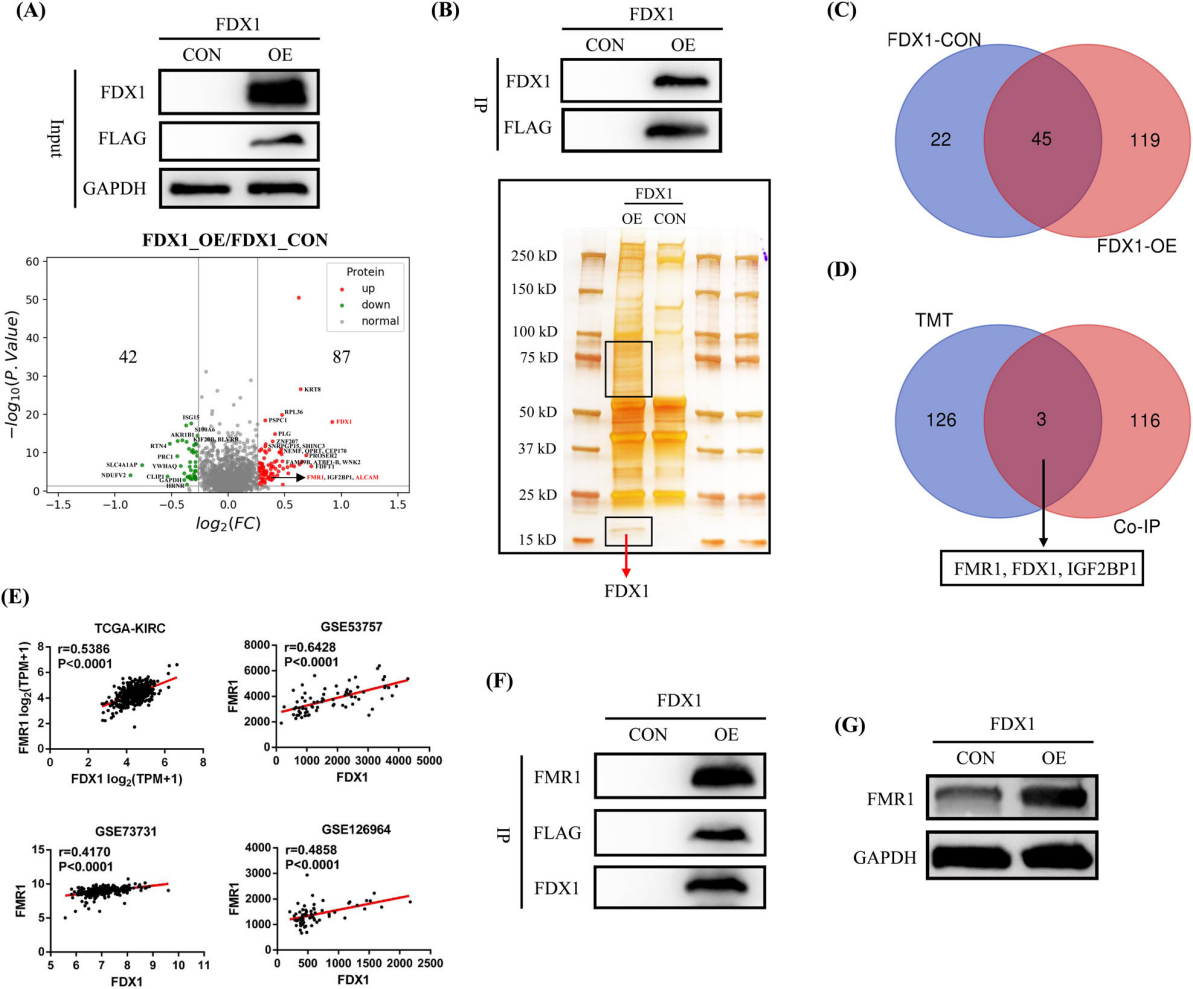
FDX1 protein binds to the FMR1 protein and promotes its expression. **A** TMT detection of FDX1-overexpressing Caki-1 cells and control cells. **B** IP experiment of FDX1 and silver staining detection. **C** Mass spectrometry detection results of differential protein bands. **D** Joint analysis of the TMT data and mass spectrometry data. **E** The correlation between FMR1 expression and FDX1 expression based on the data of TCGA-KIRC and 3 GSE datasets (GSE53757, GSE73731 and GSE126964). **F** Validation of FDX1 and FMR1 protein binding. **G** Validation of the promotion effect of FDX1 on FMR1 expression.

To explore the potential downstream molecular regulatory mechanisms of FDX1, we used TMT proteomics sequencing to detect the expression of various proteins in FDX1-overexpressing Caki-1 cells and control cells, and performed differential protein comparison analysis. Compared with those in control cells, 87 proteins were significantly upregulated and 42 proteins were markedly downregulated in FDX1-overexpressing Caki-1 cells (Fig. 4A and Supplementary Table 1). Then, we used immunoprecipitation (IP) and silver staining experiments to detect the potential proteins that bind to the FDX1 protein. First, we successfully detected the expression of FDX1 and the tag protein FLAG in FDX1-overexpressing Caki-1 cells in IP protein samples via western blot. Then, we used silver staining to detect the differential protein bands between FDX1-overexpressing Caki-1 cells and control cells. Our results indeed found multiple differential protein bands in the FDX1-overexpressing group (Fig. 4B), and mass spectrometry detection was performed on the differential protein bands to identify the possible proteins in these groups (Fig. 4C). By combining the TMT and protein mass spectrometry detection results, we preliminarily identified that FMR1 and IGF2BP1 may be potential downstream regulatory proteins of FDX1 (Fig. 4D). In addition, we used data from the TCGA-KIRC and GEO databases to analyse the correlation between FDX1 expression and the expression of FMR1 and IGF2BP1. The results showed a significant positive correlation between FDX1 expression and FMR1 expression (Fig. 4E). Finally, we validated the binding between the FDX1 and FMR1 proteins in the IP protein samples (Fig. 4F) and confirmed the upregulation of FMR1 expression in the FDX1-overexpressing cells (Fig. 4G).

### FMR1 expression is significantly decreased in ccRCC and is positively correlated with FDX1 expression

**Fig. 5:**
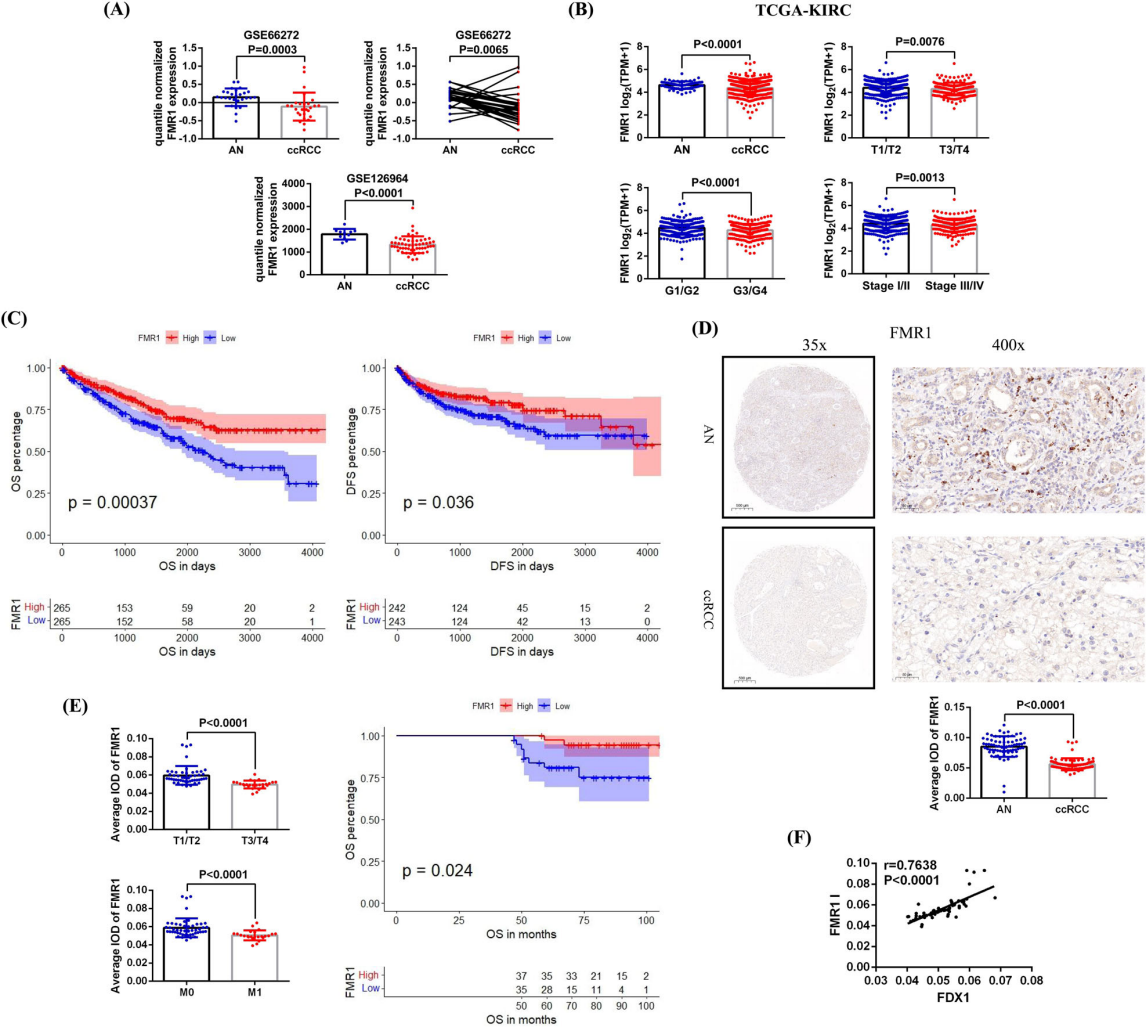
The clinical significance of FMR1 in ccRCC and its correlation with FDX1 expression. **A** Comparison of FMR1 expression between ccRCC and AN tissues based on the data of 2 GSE datasets (GSE66272 and GSE126964). **B** Comparison of FMR1 expression between ccRCC and AN tissues based on TCGA-KIRC data, and its expression differences in tumors with different pathological features. **C** Analysis of the relationships between FMR1 expression and the OS and DFS of patient based on TCGA-KIC data. **D** The expression of FMR1 in 75 pairs of ccRCC and AN tissues detected by immunohistochemistry. **E** Comparison of the differences of FMR1 expression in tumors with different pathological features and analysis of the relationship between FMR1 expression and the OS of patients. (**F**) Correlation between FMR1 expression and FDX1 expression in these 75 ccRCC paraffin sections.

We analysed the expression data of FMR1 in 2 GSE datasets (GSE66272 and GSE126964), and found that FMR1 expression was significantly lower in ccRCC than in AN tissues (Fig. 5A). Besides, we also detected a low-expression state of FMR1 in ccRCC using TCGA-KIRC data, and lower expression of FMR1 was closely associated with higher tumor stages and grades (T3/T4, G3/G4 and Stage III/IV) (Fig. 5B). The results of prognosis analysis showed that the OS and DFS of patients with low FMR1 expression were significantly shorter than those of patients with high FMR1 expression (Fig. 5C). In addition, we used IHC to detect the expression of FMR1 in 75 pairs of ccRCC and AN tissues in our center, and further analysis was carried out in combination with the clinicopathological characteristics and survival times of the patients. Our results verified that FMR1 expression was significantly reduced in ccRCC (Fig. 5D) and that decreased FMR1 expression was closely associated with higher tumor stages (T3/T4), distant metastasis (M1), and shorter OS (Fig. 5E). Finally, we conducted a correlation analysis on the expression of FDX1 and FMR1 in these 75 ccRCC tissues, and our results confirmed a significant positive correlation between FDX1 expression and FMR1 expression (Fig. 5F).

### FMR1 binds to the FDX1 protein and may regulate ALCAM expression

**Fig. 6:**
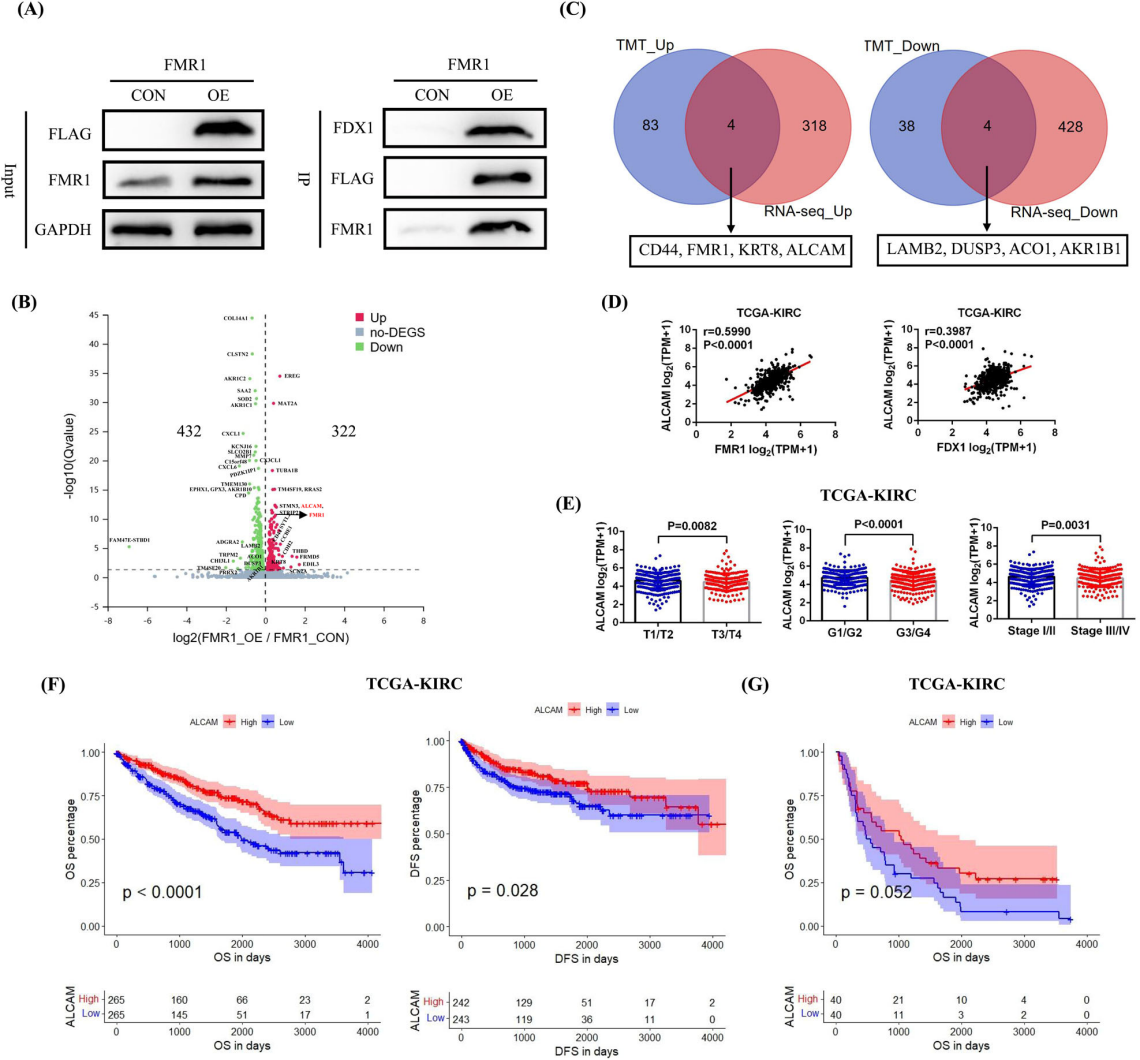
Preliminary identification of the potential downstream target molecule ALCAM that may be regulated by FDX1/FMR1. **A** Verification of binding between the FMR1 and FDX1 protein by IP experiment. **B** RNA-seq detection of FMR1-overexpressing Caki-1 cells and control cells. **C** Joint analysis of the detection data of TMT and RNA-seq. **D** The correlation between ALCAM expression and the expression of FMR1 and FDX1 based on TCGA-KIRC data. **E** Comparison of the differences of ALCAM expression in tumors with different pathological features based on TCGA-KIRC data. **F** The relationships between ALCAM expression and the OS and DFS of patients based on TCGA-KIRC data. **G** The relationships between ALCAM expression and the OS of metastatic ccRCC patients in TCGA-KIRC.

To further validate the binding between FDX1 and the FMR1 protein, we successfully overexpressed the FMR1 protein in Caki-1 cells, and our IP experiment results also confirmed that FMR1 could indeed bind to the FDX1 protein (Fig. 6A). In addition, we used RNA-seq to detect the expression of various genes in FMR1-overexpressing and control Caki-1 cells and conducted a comparative analysis. Our analysis results found that compared with those in the control cells, 322 genes were upregulated and 432 genes were downregulated in the FMR1-overexpressing Caki-1 cells (Fig. 6B and Supplementary Table 2). By combining the differential protein expression data detected by TMT and the differential gene expression data detected by RNA-seq, we found that the expression of 4 molecules (CD44, FMR1, KRT8, and ALCAM) was upregulated in both FDX1-overexpressing Caki-1 cells and FMR1-overexpressing Caki-1 cells, whereas the expression of 4 molecules (LAMB2, DUSP3, ACO1, and AKR1B1) was downregulated in both FDX1-overexpressing Caki-1 cells and FMR1-overexpressing Caki-1 cells (Fig. 6C). Then, we used the TCGA-KIRC data to compare the correlations between the expression of these differentially expressed genes and the expression of FDX1 and FMR1. Our results found that only ALCAM expression was significantly positively correlated with both FMR1 and FDX1 expression (Fig. 6D). In addition, ALCAM expression was also significantly lower in high-stage and high-grade tumors (T3/T4, G3/G4, and Stage III/IV) than in low-stage and low-grade tumors (T1/T2, G1/G2, and Stage I/II) (Fig. 6E). The prognostic analysis results suggested that patients with low ALCAM expression had shorter OS and DFS than did those with high ALCAM expression (Fig. 6F). Furthermore, patients with distant metastases were selected for prognostic analysis on the basis of the ALCAM expression data of these tumors, and the results also confirmed that patients with low ALCAM expression tended to have shorter OS (Fig. 6G).

### Validation of the expression and prognostic significance of ALCAM in clinical ccRCC samples

**Fig. 7:**
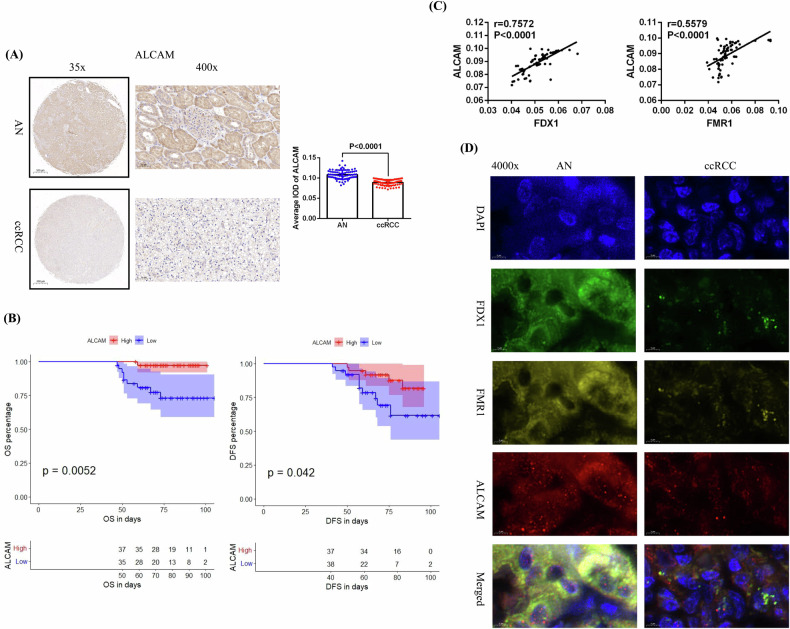
The clinical value of ALCAM in ccRCC and its correlation with FDX1 and FMR1 expression. **A** The expression of ALCAM in 75 pairs of ccRCCs and AN tissues detected by immunohistochemistry. **B** Analysis of the relationships between ALCAM expression and the OS and DFS of patients. **C** Correlations between ALCAM expression and the expression of FDX1 and FMR1 in these 75 ccRCC paraffin sections. **D** Sublocalization detection of FDX1, FMR1, and ALCAM proteins in ccRCC and AN tissues by immunofluorescence.

Similarly, we used IHC to detect ALCAM expression in these 75 pairs of ccRCC and AN tissues, and the analysis results identified that ALCAM was significantly underexpressed in ccRCC compared to the AN tissues (Fig. 7A). Prognostic analysis results also confirmed that patients with low ALCAM expression had significantly shorter OS and DFS than did those with high ALCAM expression (Fig. 7B). In addition, the correlation analysis results demonstrated that ALCAM expression was significantly positively correlated with both FDX1 and FMR1 expression in these 75 ccRCC tissues (Fig. 7C). Finally, we used immunofluorescence to detect the sublocalization of FDX1, FMR1, and ALCAM in ccRCC and AN tissues and confocal laser microscopy for image acquisition and analysis. Our results verified that both FDX1 and FMR1 were expressed mainly in the cytoplasm, while ALCAM was expressed in both the cytoplasm and nucleus, but these three proteins were colocalized in the cytoplasm (Fig. 7D).

### FDX1 may affect ALCAM expression by regulating FMR1

**Fig. 8:**
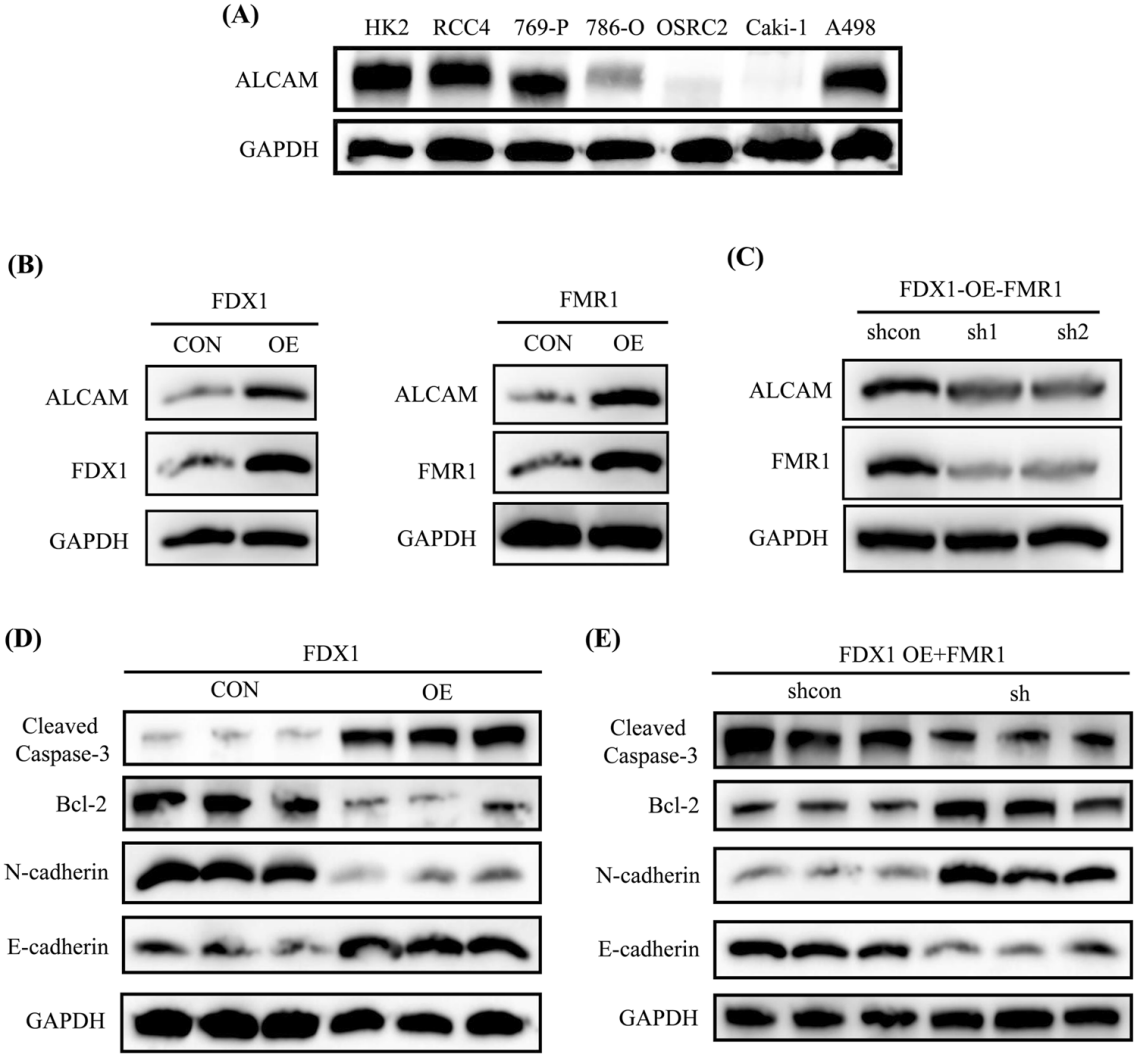
FDX1 may affect ALCAM expression by regulating FMR1 in ccRCC cells. **A** The expression of ALCAM protein in HK2 cell line and 6 ccRCC lines (RCC4, 769-P, 786-O, OSRC2, Caki-1, and A498). **B** Expression changes of ALCAM in FDX1-overexpressing Caki-1 cells and FMR1-overexpressing Caki-1 cells. **C** The effect of knocking down FMR1 expression on ALCAM expression on the basis of FDX1 overexpression. **D** The effect of FDX1 overexpression on the expression of apoptosis-related proteins Bcl-2 and Cleaved Caspase-3, and metastasis-related proteins E-cadherin and N-cadherin. **E** The effect of knocking down FMR1 expression based on the overexpression of FDX1 on the expression of Bcl-2, Cleaved Caspase-3, E-cadherin, and N-cadherin.

To clarify whether FDX1 affects ALCAM expression by regulating FMR1, we first detected the expression of the ALCAM protein in 6 ccRCC lines (RCC4, 769-P, 786-O, OSRC2, Caki-1, and A498) and compared it with its expression in the HK2 cell line. Our western blot results indicated that, compared with that in HK2 cells, ALCAM protein expression was significantly lower in 786-O, OSRC2, and Caki-1 cell lines (Fig. 8A). We also validated the promoting effects of FDX1 and FMR1 expression on ALCAM expression in FDX1-overexpressing and FMR1-overexpressing Caki-1 cell lines, respectively (Fig. 8B). Besides, the expression of ALCAM was significantly decreased after FMR1 expression was knocked down in FDX1-overexpressing Caki-1 cell lines (Fig. 8C). Moreover, we detected the expression of the apoptosis-related proteins Bcl-2 and Cleaved Caspase-3, as well as the expression of the metastasis-related proteins E-cadherin and N-cadherin, in FDX1-overexpressing and control Caki-1 cells. It was found that after FDX1 overexpression, the expression of E-cadherin and Cleaved Caspase-3 was significantly increased, whereas the expression of Bcl-2 and N-cadherin was remarkably decreased (Fig. 8D). In addition, we detected the expression of Bcl-2, Cleaved Caspase-3, E-cadherin, and N-cadherin after FMR1 expression was knocked down on the basis of FDX1 overexpression. The western blot results also validated that after knocking down FMR1 expression on the basis of FDX1 overexpression, the expression of E-cadherin and Cleaved Caspase-3 was significantly reduced, whereas the expression of Bcl-2 and N-cadherin was remarkably enhanced (Fig. 8E). Therefore, our above results demonstrated that FDX1 likely regulates the expression of ALCAM by modulating FMR1 in ccRCC cells.

### Knockdown of FMR1 expression on the basis of FDX1 overexpression reverses the inhibition of the cell phenotype caused by FDX1 overexpression in vitro

**Fig. 9:**
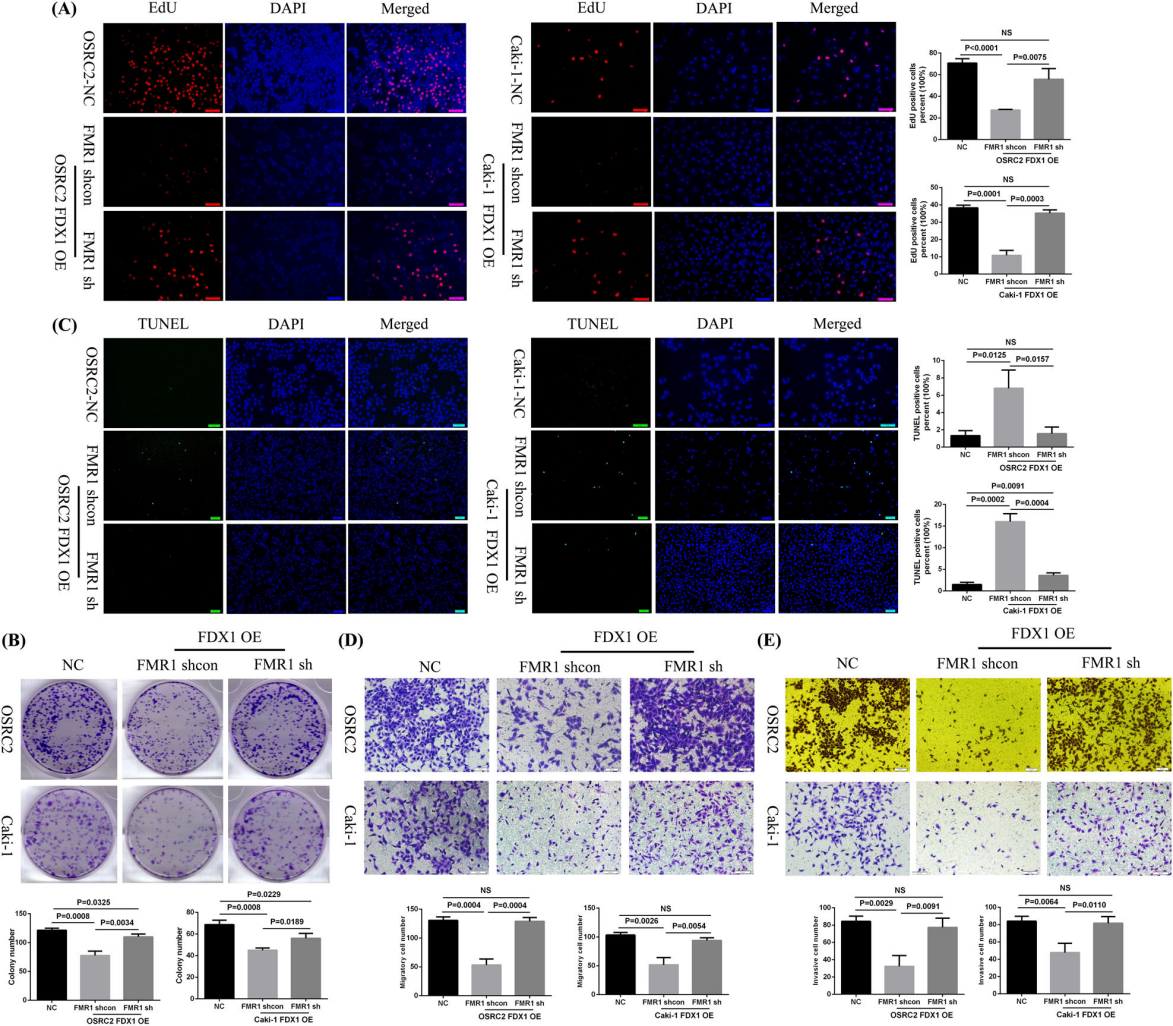
The effects of FMR1 knockdown on the phenotype of FDX1-overexpressing ccRCC cells in vitro. **A**–**E** The effect of knockdown of FMR1 expression on cell proliferation (EdU), cell growth (clone formation assay), cell apoptosis (TUNEL), cell migration, and cell invasion, respectively. NC negative control, NS no statistical significance.

To determine whether FDX1 affects the ccRCC cell phenotype by regulating FMR1, the immunofluorescence EdU method and plate cloning experiment, the immunofluorescence TUNEL method, the cell migration experiment, and the cell invasion experiment were utilised to detect the effect of FMR1 knockdown on cell proliferation, apoptosis, cell migration, and cell invasion, respectively. Our experimental results determined that after knocking down FMR1 expression on the basis of FDX1 overexpression, the number of cell proliferation was remarkably increased (Fig. 9A, B), cell apoptosis was restrained (Fig. 9C), and the cell migration and invasion ability was significantly enhanced (Fig. 9D, E).

### FDX1 overexpression inhibits the growth and metastasis of ccRCC cells in vivo

**Fig. 10:**
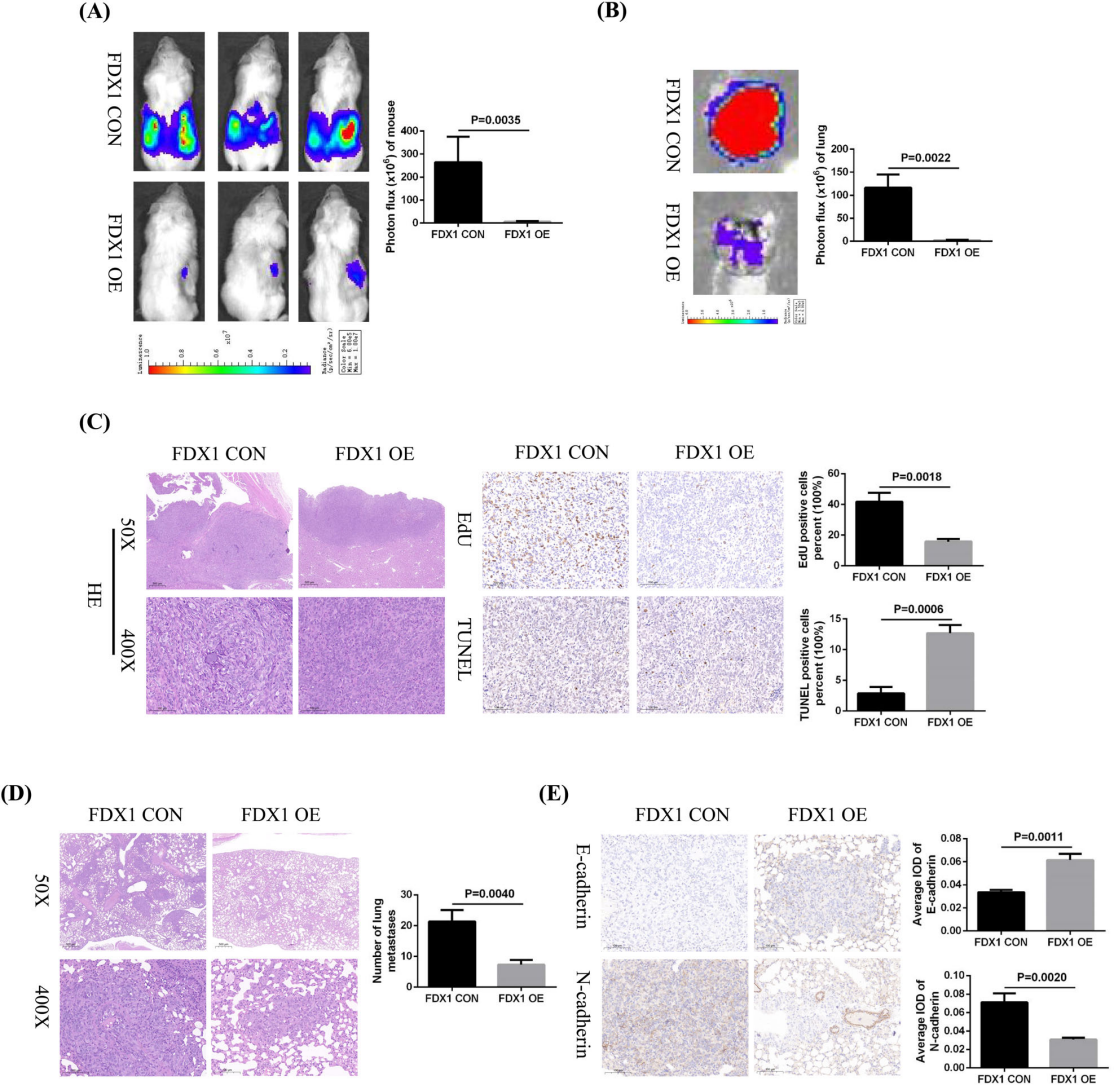
FDX1 overexpression inhibits the growth and metastasis of ccRCC cells in vivo*.* **A** Bioluminescence imaging of mouse renal tumors in the FDX1 overexpression and the control groups. **B** In vitro imaging of mouse lung metastases in FDX1 overexpression and the control groups. **C** H&E staining and immunohistochemical staining of EDU and TUNEL expression in mouse renal tumors. **D** H&E staining of mouse lung metastases. **E** Immunohistochemical staining of E-cadherin and N-cadherin expression in mouse lung metastases.

To further clarify the inhibitory effect of FDX1 overexpression on the ccRCC phenotype, we successfully constructed a mouse orthotopic renal tumor growth model. FDX1-overexpressing or its control caki-1 cells were injected into the renal capsules of immunodeficient mice, and the growth and metastasis of renal tumors in the mice were observed regularly through bioluminescence imaging. The bioluminescence imaging results confirmed that the growth rate of renal tumors in the FDX1-overexpressing group was significantly lower than that in the control group (Fig. 10A), and the in vitro lung imaging results indicated that the tumor lung metastasis rate in the FDX1-overexpressing group was also lower than that in the control group (Fig. 10B). H&E staining was used to detect the tissue structure of mouse renal tumors and lung metastases. Immunohistochemical staining was utilised to detect the expression of EdU and TUNEL in the paraffin sections of renal tumors, as well as the expression of E-cadherin and N-cadherin in the paraffin sections of lung metastases. Our results indicated that, compared with that in the control group, EdU expression was significantly reduced and TUNEL expression was remarkably increased in the FDX1-overexpressing group (Fig. 10C). The H&E staining results showed that the number of lung metastases in the FDX1-overexpressing group was significantly lower than that in the control group (Fig. 10D). Moreover, compared with that in the control group, the expression of E-cadherin in the lung metastases of the FDX1-overexpressing group was significantly greater, whereas the expression of N-cadherin was remarkably decreased (Fig. 10E). Thus, these results verified the inhibitory effect of FDX1 overexpression on the proliferation and metastasis of ccRCC cells in mice models.

### FDX1 suppresses the growth and metastasis of ccRCC by regulating FMR1 in vivo

**Fig. 11:**
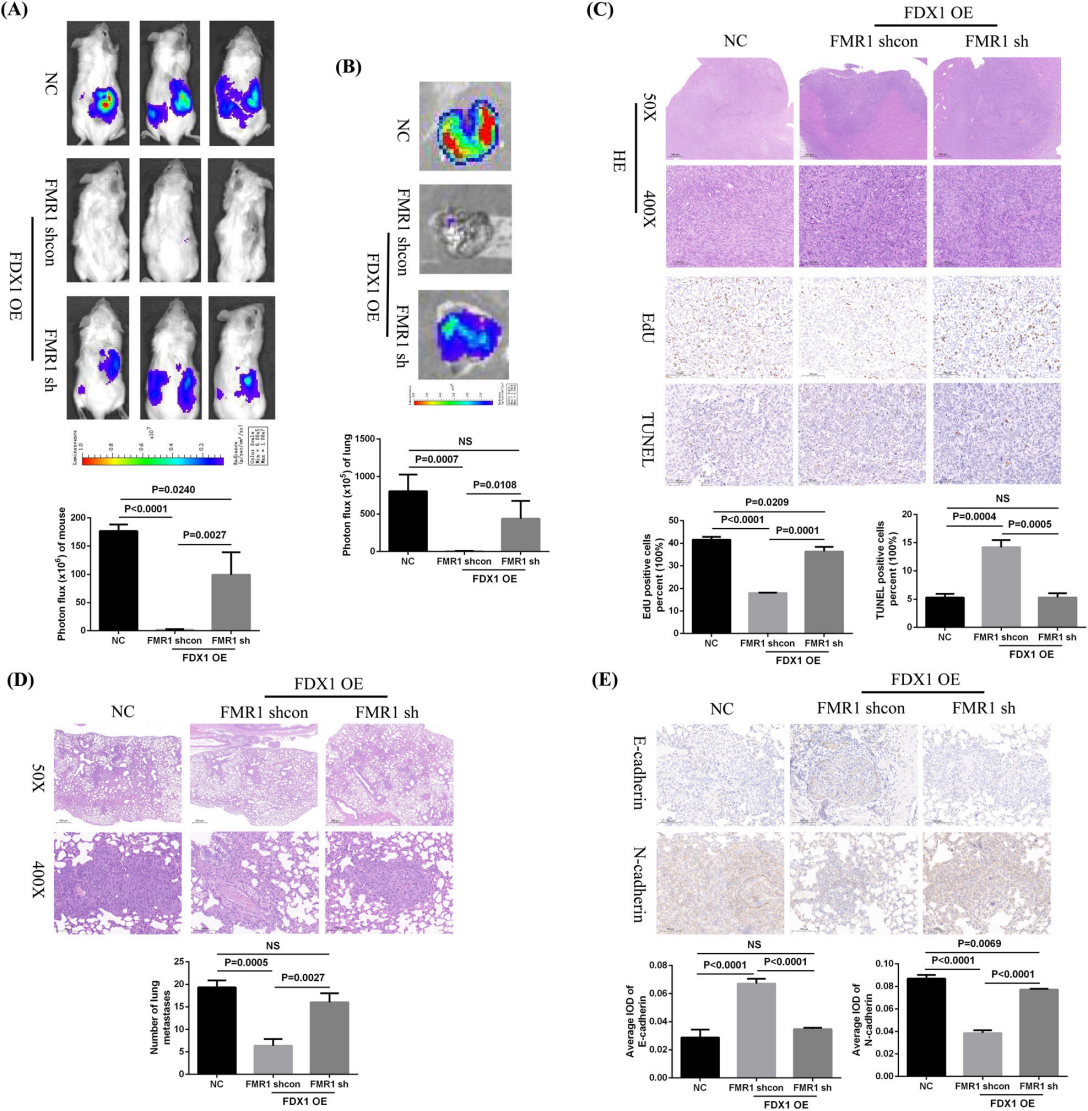
FDX1 overexpression suppresses the growth and metastasis of ccRCC cells by regulating FMR1 in vivo. **A** Bioluminescence imaging of mouse renal tumors in FMR1 knockdown group based on FDX1 overexpression and the FDX1-overexpressing group. **B** In vitro imaging of mouse lung metastases in FMR1 knockdown group based on FDX1 overexpression and the FDX1-overexpressing group. **C** H&E staining and immunohistochemical staining of EDU and TUNEL expression in mouse renal tumors. **D** H&E staining of mouse lung metastases. **E** Immunohistochemical staining of E-cadherin and N-cadherin expression in mouse lung metastases. NC negative control, NS no statistical significance.

Similarly, Caki-1 cells that knocked down FMR1 on the basis of FDX1 overexpression and FDX1-overexpressing Caki-1 cells were also injected into the renal capsules of mice, and the growth and metastasis of renal tumors in the mice were observed regularly through bioluminescence imaging. Bioluminescence imaging results proved that the growth rate of renal tumors in the FMR1-knockdown group was significantly faster than that in FDX1-overexpressing group (Fig. 11A), and in vitro lung imaging results also suggested that the tumor lung metastasis rate in the FMR1-knockdown group was also faster than that in the FDX1 overexpression group (Fig. 11B). The immunohistochemical staining results indicated that after knocking down the expression of FMR1 based on FDX1 overexpression, EdU expression in renal tumors was significantly increased, whereas TUNEL expression was markedly decreased (Fig. 11C). The H&E staining results also shown that there were more lung metastases in the FMR1-knockdown group than in the FDX1-overexpressing group (Fig. 11D). Moreover, compared with that in the FDX1-overexpressing group, E-cadherin expression in the lung metastases of the FMR1-knockdown group was significantly lower, whereas N-cadherin expression was remarkably upregulated (Fig. 11E). Therefore, the above results fully demonstrated that FDX1 suppressed the proliferation and metastasis of ccRCC cells by regulating FMR1 in mice models.

## Discussion

FDX1, located on chromosome 11q22, encodes a low molecular weight protein containing iron–sulfur (Fe–S) clusters as REDOX active groups, which can transfer electrons from NADPH to mitochondrial cytochrome P450 [14]. The electron transport chain process of mitochondria is closely related to many metabolic related pathways and inflammatory reactions, which play essential roles in the occurrence and development of cancer [15]. Recently, many studies have pointed out that FDX1 is a direct target of copper-dependent cell death and have demonstrated its potential value in tumor therapy. For example, FDX1 inhibited the adaptive ability of tumor cells to proteasome inhibition, which may enhance the clinical application of proteasome inhibitors [8]; enzalutamide and copper ionophorets could synergistically induce copper poisoning death in cells and reduce drug resistance, which may provide a promising therapeutic strategy for castration-resistant prostate cancer [16]; increased expression of adrenomedullin activated the p38/MAPK signaling pathway and promoted the phosphorylation and nuclear uptake of Forkhead box O3, thereby suppressing the transcription of FDX1 and cellular copper deposition and promoting chemotherapy resistance in ccRCC [17]. However, the specific role of FDX1 in ccRCC still needs further clarification.

In this study, we further validated the low-expression characteristic of FDX1 in ccRCC, and its reduced expression could predict adverse tumor pathological conditions and shorter OS and DFS of patients. Moreover, our in vitro and in vivo experiments have fully validated the inhibitory effects of FDX1 overexpression on the proliferation and metastasis of ccRCC cells, indicating its role as a tumor suppressor gene in ccRCC. Mechanistically, we found that FDX1 could bind to the FMR1 protein and promote its expression. The fragile X mental retardation 1 (FMR1) gene is located on human chromosome Xq27.3 and encodes the FMR1 protein (also known as FMRP) [18], which is an RNA-binding protein that can regulate RNA variable cutting, mRNA stability and translation [19]. Many studies have indicated that the FMR1 protein plays a crucial role in the progression of various tumors. For instance, FMR1 bound to CCAR1 mRNA and regulated CCAR1 post-transcriptionally to activate the Wnt signaling pathway and promote the stemness of hepatocellular carcinoma cells [20]; the increased expression of FMR1 in melanoma promoted melanoma cell invasion and metastasis by regulating the mRNA expression of its downstream targets [21]; and the FMR1 protein could regulate the key molecules involved in epithelial mesenchymal transition (EMT) post-transcriptionally by interacting with relevant mRNAs, thereby promoting breast cancer progression [22].

In the current study, we validated the low-expression status of FMR1 in ccRCC, and its decreased expression was closely associated with higher tumor stages, metastasis, and poor prognosis of patients. Besides, we found that FDX1 may affect ALCAM expression by regulating FMR1 by RNA-seq and western blot. ALCAM (activated leukocyte cell adhesion molecule), also known as CD166, plays a crucial role in mediating the adhesion of cancer cells [23]. ALCAM has been shown to promote the adherence, proliferation, and growth of cells in multiple tumors, such as breast cancer and glioma [24, 25]. For example, miR-483-5p exerted its metastasis-promoting function by directly targeting ALCAM, a putative metastasis suppressor, after being activated by the WNT/β-catenin signaling pathway [26]. In addition, increased ALCAM expression was strongly associated with poor prognosis of gastric and pancreatic cancer patients [27, 28]. However, in breast, thyroid and prostate cancers, a high-expression level of ALCAM was a favorable prognostic factor [29–31].

During this study, we confirmed that ALCAM expression was significantly reduced in ccRCC and that decreased ALCAM expression predicted shorter OS and DFS of patients. ALCAM expression was significantly positively correlated with the expression of FDX1 and FMR1. Besides, the protein expression of FDX1, FMR1, and ALCAM was colocalized in the cytoplasm of ccRCC cells, but ALCAM was also expressed in the nucleus. Moreover, our western blot results also identified that after FDX1 overexpression, ALCAM expression was significantly increased, while the expression of Cleaved Caspase-3 and E-cadherin was increased and the expression of Bcl-2 and N-cadherin was decreased. However, knocking down FMR1 expression on the basis of FDX1 overexpression remarkably suppressed the increased expression of ALCAM, while the expression of Cleaved Caspase-3 and E-cadherin was reduced, and the expression of Bcl-2 and N-cadherin was upregulated. Therefore, our above results demonstrated that FDX1 may affect the expression of ALCAM, apoptosis-related proteins, and metastasis-related proteins by regulating FMR1 in ccRCC.

EMT is a mechanism associated with cancer metastasis, and the migration ability of tumor cells can enhanced through EMT induction. During the EMT process, the protein expression of N-cadherin is increased, whereas E-cadherin protein expression is decreased, thereby mediating EMT-induced tumor invasion. Therefore, anti-tumor drugs that target the EMT process can reduce cancer invasion and improve the sensitivity of cancer cells to drug therapy [32]. Our research results confirmed that FDX1 overexpression promoted the expression of E-cadherin and inhibited the expression of N-cadherin, which suggested that FDX1 may suppress the EMT process in ccRCC. M6A modification is a common form of internal RNA modification closely related to gene and protein regulation, and previous studies have proved that epigenetic modifications, including M6A modification, are closely related to the regulation of EMT [33]. Currently, FMR1 is known to be involved in regulating cancer progression as a novel m6A reader [34]. Therefore, FMR1 may affect the EMT process through regulating the m6A modification of its downstream molecules in cancer, and the regulation of FMR1 on the M6A modification of EMT-related molecules deserves further investigation.

In addition, our results have validated that FDX1 may affect the expression of ALCAM by regulating FMR1. Previous studies have suggested that ALCAM is closely related to tumor-infiltrating immune cells and tumor immunotherapy. For instance, macrophages with high ALCAM expression colocalized with depleted CD8+ T cells in the tumor microenvironment to promote T cell depletion, and the inhibition of HIF-1ɑ could reduce the expression of ALCAM in macrophages and deplete CD8+ T cells, thereby improving the effect of immunotherapy by enhancing the anti-tumor function of T cells [35]. Conventional type 1 dendritic cells (cDC1) were key regulatory factors in anti-tumor T cell responses, and ALCAM-mediated interactions between cDC1-CD8 T cells were inhibited in advanced lung tumors [36]. Thus, further exploration of the relationship between the regulation of FMR1 by FDX1 and the tumor immune microenvironment will contribute to a deeper understanding of tumor immunotherapy.

Furthermore, this study has certain limitations, such as the specific binding sites of the FDX1 and FMR1 proteins and the potential specific mechanisms by which FDX1 promotes FMR1 expression, which need to be further clarified. In addition, the specific regulatory mechanisms by which FMR1 affects ALCAM transcription, as well as the relationship between copper death and this regulatory pathway in ccRCC, deserve further investigation. Finally, more evidence is needed to support whether FDX1 only affects the expression of ALCAM and other downstream molecules by regulating FMR1.

Nevertheless, our results fully confirmed that FDX1 exhibited a low-expression pattern in ccRCC, and its reduced expression was associated with higher tumor stages and grades. Reduced FDX1 expression was related to shorter OS and DFS in ccRCC patients, indicating that FDX1 could serve as a biomarker for predicting the prognosis of ccRCC patients. Mechanistically, FDX1 inhibited the growth and metastasis of ccRCC cells by binding to the FMR1 protein and promoting its expression. Therefore, our results suggest that FDX1 acts as a tumor suppressor in ccRCC. However, to provide a solid theoretical basis for the development of new drugs for ccRCC treatment, further research is needed to clarify the specific mechanisms by which FDX1 regulates FMR1 and the effects of FMR1 on its downstream pathways, including ALCAM.

## Materials and methods

### Ethics statement

This study was performed in accordance with the Declaration of Helsinki and approved by the Institutional Ethical Board of the First Affiliated Hospital, Zhejiang University School of Medicine (Ethical approval number: 2024 Research No. 0921). Informed consent forms signed by each patient or their immediate family member were obtained. All animal experimental procedures used in this study were approved by the Laboratory Animal Ethics Committee.

### Bioinformatics data collection

The mRNA expression data of FDX1, FMR1 and ALCAM in ccRCC and adjacent normal (AN) tissues were downloaded from The Cancer Genome Atlas Program (TCGA) and Gene Expression Omnibus(GEO) databases. Kidney Renal Clear Cell Carcinoma (KIRC) data from the TCGA and 4 GSE datasets (GSE40435, GSE66272, GSE105261, and GSE126964) from the GEO were used in this study. The clinicopathological characteristics and survival times of these ccRCC patients were also obtained.

### Clinical sample collection

The paraffin sections of 75 pairs of ccRCCs and their AN tissues were obtained from the Department of Urology, the First Affiliated Hospital, Zhejiang University School of Medicine. In addition, the clinicopathological information of these tumors and the survival time information of the patients were also obtained.

### Cell culture

This study utilised a total of 8 cell lines, including HEK-293T, HK2 and 6 ccRCC cell lines (RCC4, 769-P, 786-O, OSRC2, Caki-1, and A498). All of these cell lines were purchased from the American Type Culture Collection (ATCC). The cell culture medium was DMEM containing 10% FBS. In addition, we constructed an FDX1 overexpression plasmid, FMR1 overexpression plasmid, and FMR1 knockdown plasmid. The sequences of the shRNAs targeting FMR1 were as follows: sh1-CGAGATTTCATGAACAGTTTA; sh2-GCGTTTGGAGAGATTACAAAT. FDX1-overexpressing, FMR1-overexpressing, and FMR1-knockdown lentiviruses were packaged using a 3-plasmid system, and Lipofectamine 3000 (Thermo Fisher, USA) was used as the transfection reagent.

### Immunohistochemistry (IHC)

The protein expression levels of FDX1, FMR1, and ALCAM in the paraffin sections of 75 pairs of ccRCCs and their AN tissues in our center were detected via immunohistochemical staining. In addition, the protein expression of E-cadherin and N-cadherin in mouse lung metastases was also detected. The specific primary antibodies used were as follows: anti-FDX1 (12592-1-AP, Proteintech, 1:100), anti-FMR1 (13755-1-AP, Proteintech, 1:400), anti-ALCAM (RT1029, HUABIO, 1:100), anti-E-cadherin (ab40772, Abcam, 1:500), and anti-N-cadherin (ab19348, Abcam, 1:1000). The images were collected under a microscope and the average integrated optical density (IOD) of the protein expression (FDX1, FMR1, ALCAM, E-cadherin, and N-cadherin) was calculated via IPP (Image Pro Plus 6.0) software.

### Western blot

First, total protein was extracted from ccRCC cells using RIPA lysis buffer and subjected to protein quantification analysis. After electrophoresis, membrane transfer and blocking, primary antibody incubation was performed at 4 °C overnight. The primary antibodies used were as follows: anti-FDX1 (12592-1-AP, Proteintech, 1:1000), anti-FMR1 (13755-1-AP, Proteintech, 1:1000), anti-FLAG (F3165, Sigma-Aldrich, 1:1000), anti-ALCAM (RT1029, HUABIO, 1:1000), anti-Cleaved-Caspase 3 (9661S, CST, 1:1000), anti-Bcl-2 (12789-1-AP, Proteintech, 1:2000), anti-E-cadherin (1:10000; Abcam, ab40772), anti-N-cadherin (1:5000, Abcam, ab76011), and GAPDH (60004-1-Ig, Proteintech, 1:20000). The next day, the membrane was incubated with the secondary antibody at room temperature for 2 h, and enhanced chemiluminescence was performed after the PVDF membrane was washed.

### Cell proliferation detection

EdU (5-Ethynyl-2′-deoxyuridine) was used as a cell proliferation marker, and the effects of FDX1 overexpression and FMR1 knockdown on the proliferation ability of ccRCC cells were detected in vitro and in vivo via the cell fluorescence method and tissue DAB staining methods, respectively. The reagent kits (EdU-647 Cell Proliferation Detection Kit and EdU Cell Proliferation Detection Kit (DAB method)) used were purchased from Beyotime Biotechnology Company. In addition, a cell plate colony formation experiment was also used to test the growth ability of the cells. A moderate number of OSRC2 and Caki-1 cells were inoculated into well plates, and the cells in the well plate were stained with 0.5% crystal violet after approximately 2 weeks of cultivation. After washing thoroughly, the image was scanned for subsequent analysis.

### Cell apoptosis detection

A TUNEL cell apoptosis detection kit with a fluorescence method and DAB staining method were used to detect cell apoptosis in cell crawling slices and paraffin-embedded tissue, respectively. The reagent kits (one step TUNEL cell apoptosis detection kit (green fluorescence) and 3,3′-diaminobenzidine (DAB) used were also purchased from Beyotime Biotechnology Company.

### Detection of cell migration and invasion ability

To test the migration ability of the cells, 200 μL of serum-free cell culture medium mixed with a moderate amount of OSRC2 and Caki-1 cells was inoculated into the upper chamber, and 700 μL of cell culture medium containing 10% FBS was added to the lower chamber. After 2 days of cultivation, the cells below the membrane surface of the lower chamber were washed with distilled water and stained with 0.5% crystal violet. After washing thoroughly, images were collected under an inverted microscope for subsequent analysis. In addition, the method for detecting cell invasion ability was the same as that described above, except that the upper chamber was coated with Matrigel.

### TMT (tandem mass tag) test

Caki-1 cells overexpressing FDX1 and control Caki-1 cells were subjected to TMT-based quantitative proteomics, which was performed by Beijing Liuhe Huada Gene Technology Co., Ltd. The detailed detection method has been described previously [37]. Through ANOVA analysis of variance, proteins with *P* < 0.05 and a difference factor greater than 1.2 were selected as differential proteins.

### Co-Immunoprecipitation (Co-IP) assay

The Co-IP experiments were used to search for and validate other potential proteins bound to the FDX1 protein. First, the protein supernatant of the cells was extracted using IP lysis buffer, and a portion was retained as the input protein sample. Anti-Flag M2 magnetic beads (M8823, Millipore, 1:40) were added to the remaining protein supernatant and incubated overnight at 4 °C. The next day, the magnetic beads were washed 3 times with IP lysis buffer, 100 μl 2× protein loading buffer was added, the mixture was boiled at 100 °C for 5 min, the protein loading buffer was collected and the magnetic beads were discarded. The potential binding proteins were subsequently identified through protein electrophoresis, silver staining (P0017S, Beyotime), mass spectrometry (performed by Beijing Liuhe Huada) and western blot.

### RNA-sequencing (RNA-seq)

Caki-1 cells which FMR1 expression was knocked down on the basis of FDX1 overexpression and FDX1-overexpressing Caki-1 cells were tested via RNA-seq, which was performed by Beijing Liuhe Huada Gene Technology Co., Ltd using the BGISEQ platform. Differential gene analysis was performed via the DEseq2 method.

### Immunofluorescence

On the first day, the tissue paraffin sections were subjected to FDX1 (12592-1-AP, Proteintech, 1:200) antibody incubation at 4 °C overnight after dewaxing, antigen repair and serum blocking. The next day, the samples were washed with PBS, incubated with secondary antibody (green), washed again, blocked with serum and incubated with FMR1 (13755-1-AP, proteintech, 1:100) antibody at 4 °C overnight. On the third day, washed with PBS, incubated with secondary antibody (yellow), washed again, blocked with serum and incubated with ALCAM (RT1029, HUABIO, 1:100) antibody at 4 °C overnight. On the fourth day, the samples were washed with PBS, incubated with secondary antibody (red), and stained with DAPI after washing. The sections were washed again, and images were collected under a laser confocal microscope.

### Orthotopic renal tumor mouse model

Fifteen 6-week-old B-NDG immunodeficient mice were purchased from Beijing Biocytogen Company and randomly divided into five groups, with three mice in each group. Fresh cell culture medium mixed with approximately one million viable FDX1-overexpressing Caki-1 cells and control cells, as well as Caki-1 cells with FMR1 knockdown on the basis of FDX1-overexpressing cells and control cells, was injected into the right renal capsule of each mouse. Bioluminescence imaging was performed periodically via a bioluminescence imaging method that has been described in detail in previous studies [38]. All the mice were injected with EdU (50 mg/kg) peritoneally two hours before being killed for subsequent EdU immunostaining. After the mice were killed, the lung tissue was first removed intact and immediately subjected to in vitro imaging to examine the lung metastasis of renal tumors. Fresh renal tumor tissues and lung tissues were partially stored in liquid nitrogen and partially embedded in paraffin. All animal experimental procedures were approved by the animal ethics committee of the First Affiliated Hospital, Zhejiang University School of Medicine. No blinding performed for the animal studies.

### Statistical analyses

The differences between continuous variables were tested via Student’s *t*-test and the non-parametric Mann–Whitney method. Survival curve analysis was performed via the Kaplan–Meier method and log-rank tests. The correlation between the expression of two molecules was analysed via the Pearson method. All the statistical tests were two-sided tests, and a *P* value < 0.05 was regarded as a significant difference.

## Supplementary information

Supplementary Table Legends

Supplementary Table 1

Supplementary Table 2

Full and uncropped western blots

## Data Availability

All the data analysed and generated in this study are included in this manuscript.
