# Supplementary material for: FDX1 overexpression inhibits the growth and metastasis of clear cell renal cell carcinoma by upregulating FMR1 expression
Source: Cell Death Discov. 2025 Mar 21;11:115. doi: 10.1038/s41420-025-02380-5 (PMC11928736; doi:10.1038/s41420-025-02380-5)
Supplement: Supplementary file 1 — Supplementary Table Legends [file 41420_2025_2380_MOESM1_ESM.docx]

**Supplementary Table**

**Supplementary Table 1:** Differentially expressed proteins in Figure 4A

**Supplementary Table 2:** Differentially expressed mRNAs in Figure 6B
