# Supplementary material for: FDX1 overexpression inhibits the growth and metastasis of clear cell renal cell carcinoma by upregulating FMR1 expression
Source: Cell Death Discov. 2025 Mar 21;11:115. doi: 10.1038/s41420-025-02380-5 (PMC11928736; doi:10.1038/s41420-025-02380-5)
Supplement: Supplementary file 2 — Supplementary Table 1 [file 41420_2025_2380_MOESM2_ESM.pdf]

Supplementary Table 1: Differentially expressed proteins in Figure 4A

Protein Accession	Gene Name	log2 (OE / CON)	P_value
P10109	'FDX1'	0.919	9.23E-19
A0A1W2PQ47	'FDFT1'	0.733	0.000000314
Q86WR7	'PROSER2'	0.687	5.17E-10
B2R4I8		0.652	0.00000002
P05787	'KRT8'	0.642	2.29E-27
Q53GF0		0.629	0.000000113
B2RA03		0.623	3.37E-51
A0A024R9G4	'FAM49B'	0.583	0.000000355
Q15912	'ATBF1-B'	0.556	0.000000219
B2R6U9		0.531	0.0000245
Q9Y3S1	'WNK2'	0.523	0.000000564
Q59GM9		0.484	0.00000128
K4HSH7		0.483	0.018527778
B3KT21		0.479	1.61E-08
Q9Y3U8	'RPL36'	0.476	1.14E-20
O60524	'NEMF'	0.472	2.58E-10
Q15274	'QPRT'	0.469	1.25E-11
Q5SW79	'CEP170'	0.464	5.87E-11
Q6NUL7	'SPTLC1'	0.454	0.000000263
Q14139	'UBE4A'	0.452	6.78E-11
Q96F88	'POP1'	0.445	0.00000645
A0A0S2Z5U7	'DIABLO'	0.433	0.0000808
Q6UWP7	'LCLAT1'	0.428	1.14E-08
A2A3R6	'RPS6'	0.417	0.00000137
A0A0F7G8J1	'PLG'	0.411	1.27E-15
O43819	'SCO2'	0.409	0.000000029
A0A087WUB9	'CTNNBL1'	0.405	0.00021261
B4DUC8	'MTAP'	0.395	0.000000443
X6R4W8	'ZNF207'	0.394	1.13E-13
A8KAG1		0.393	0.000610303
A0A0A0MTN0	'CUL2'	0.391	0.000028
O76021	'RSL1D1'	0.383	0.001357899
Q10713	'PMPCA'	0.381	0.000000304
A0A024R4U3	'TTLL12'	0.380	6.55E-08
P04259	'KRT6B'	0.377	0.0000218
Q00587	'CDC42EP1'	0.375	0.00000176
A0A0S2Z3H6	'CLPTM1'	0.374	0.000888394
O00231	'PSMD11'	0.374	0.0000746
A0A024R9J6	'C8orf36'	0.362	1.9E-11
Q9H2W6	'MRPL46'	0.361	0.000766436

Q9NPJ8	'NXT2'	0.358	0.000269067
Q8N9T2	'NKAPP'1	0.353	0.000000162
Q13740	'ALCAM'	0.349	0.000222282
A0A2Z6ATB6	'DBN1'	0.338	0.0000138
A0A087X0H9	'RBM26'	0.337	0.00000528
Q59EL4		0.335	8.52E-11
Q9HDC9	'APMAP'	0.335	0.0000367
A8MWD9	'SNRPGP15'	0.330	5.26E-13
O14910	'LIN7A'	0.329	0.0000102
Q8WXF1	'PSPC1'	0.328	3.49E-19
B4DEH1		0.328	0.000805321
A6QKW0	'SHINC3'	0.327	2.25E-12
P16070	'CD44'	0.325	0.0000481
Q9BVI4	'NOC4L'	0.324	0.007042937
P10599	'TXN'	0.322	3.94E-11
Q9UHX1	'PUF60'	0.318	0.00000752
A0A3B3IU46	'RAMACL'	0.316	0.0000156
A0A024R7B7	'CDC37'	0.316	2.77E-09
A0A804HI25	'DHCR7'	0.316	0.004084459
A8K5K0		0.316	0.0000709
Q9NZI8	'IGF2BP1'	0.313	0.0000368
A0A024RB01	'ITGA5'	0.309	0.00000501
P84157	'MXRA7'	0.308	0.0000011
P12004	'PCNA'	0.308	0.000000387
P25787	'PSMA2'	0.305	0.000610552
A0A024R4N0	'hCG_1640809'	0.301	8.62E-08
A8K4I8		0.300	0.003467109
A0A024R6Q1	'EIF5'	0.300	0.000000294
A8K0R1		0.297	0.0000135
P53618	'COPB1'	0.297	0.00056529
Q9P287	'BCCIP'	0.296	0.036355979
Q59ES3		0.295	5.91E-08
A0A024R718	PBEF1'	0.294	1.03E-11
P28074	'PSMB5'	0.290	6.05E-08
A8K666		0.290	0.0000625
Q562L9	'ACT'	0.289	0.000000939
Q8WXX5	'DNAJC9'	0.287	6.68E-08
A0A1W2PPR6	'PIGT'	0.285	0.004926958
A0A2R8Y6Y7	'SUCLA2'	0.281	0.000000997
Q12788	'TBL3'	0.277	3.22E-11
Q53F64		0.276	0.00000399
O15230	'LAMA5'	0.274	0.00000731
B2R7U4		0.274	4.58E-08

Q92968	'PEX13'	0.271	0.000000739
M0R2N5	'TECR'	0.268	0.00010786
Q06787	'FMR1'	0.268	0.000341737
Q9Y3B2	'EXOSC1'	0.267	0.014516789
P06703	'S100A6'	-0.271	3.27E-15
Q59EN5		-0.274	6.64E-10
P30043	'BLVRB'	-0.281	5.76E-13
B4DPP6		-0.284	5.46E-11
Q8TDJ5	'TFG/ALK fusion'	-0.287	0.00000514
Q96Q89	'KIF20B'	-0.288	3.98E-13
Q53FE5		-0.288	0.000000349
B7Z525		-0.290	0.000277181
Q92930	'RAB8B'	-0.290	0.000869228
Q59HE3		-0.290	0.000746204
A8JZY9		-0.291	2.39E-08
A0A8I5KWQ7		-0.292	0.000134717
B2R4D5		-0.297	0.00000622
Q9Y230	'RUVBL2'	-0.302	1.08E-12
O75367	'MACROH2A1'	-0.302	0.0000352
V9HW90	'HEL-75'	-0.304	0.000161401
B5BUI8	'DUSP3'	-0.308	9.62E-11
P62318	'SNRPD3'	-0.313	1.64E-11
A0A5K1VW95	'MDH1'	-0.318	0.000733899
P21399	'ACO1'	-0.324	4.91E-11
P05161	'ISG15'	-0.327	2.42E-18
O60437	'PPL'	-0.329	0.00000322
P55268	'LAMB2'	-0.333	0.000614388
Q7RTV0	'PHF5A'	-0.336	0.000000028
A0A0S2Z430	'PCK2'	-0.336	0.000114917
Q53F55		-0.340	0.00000039
A0A384P5U2		-0.349	1E-11
Q9UHL4	'DPP7'	-0.351	0.000223063
D3DQ70	'SERBP1'	-0.360	0.000197467
Q86YZ3	'HRNR'	-0.366	0.021395143
A0A140VJW2		-0.368	1.27E-13
Q9NZY4		-0.373	6.9E-18
P04406	'GAPDH'	-0.388	0.001124297
B2RCJ6		-0.390	0.0000218
P15121	'AKR1B1'	-0.405	5.11E-14
P27348	'YWHAQ'	-0.421	0.000000289
E5KNL5		-0.446	8.76E-14
A0A024RC67	'PRC1'	-0.452	8.19E-10
Q9NQC3	'RTN4'	-0.517	4.77E-13

P30622	'CLIP1'	-0.535	0.0001396
Q9BWU0	'SLC4A1AP'	-0.761	0.000000157
E7EPT4	'NDUFV2'	-0.862	0.0000785
