# Supplementary material for: FDX1 overexpression inhibits the growth and metastasis of clear cell renal cell carcinoma by upregulating FMR1 expression
Source: Cell Death Discov. 2025 Mar 21;11:115. doi: 10.1038/s41420-025-02380-5 (PMC11928736; doi:10.1038/s41420-025-02380-5)
Supplement: Supplementary file 3 — Supplementary Table 2 [file 41420_2025_2380_MOESM3_ESM.pdf]

Supplementary Table 2: Differentially expressed mRNAs in Figure 6B

Gene ID	Gene Symbol	log2 (OE / CON)	P_value
10085	'EDIL3'	1.723	0.006662423
84978	'FRMD5'	1.596	3.53E-04
7056	'THBD'	1.356	2.54E-04
6326	'SCN2A'	1.300	0.017691574
22989	'MYH15'	0.913	0.026398722
1000	'CDH2'	0.864	2.52E-04
147372	'CCBE1'	0.762	2.14E-06
3866	'KRT15'	0.743	0.030026888
2069	'EREG'	0.737	3.48E-35
79937	'CNTNAP3'	0.711	0.003480932
94274	'PPP1R14A'	0.695	1.38E-04
54843	'SYTL2'	0.688	9.86E-09
1004	'CDH6'	0.669	2.39E-05
4118	'MAL'	0.655	0.017566307
84632	'AFAP1L2'	0.640	1.20E-05
1152	'CKB'	0.628	0.047833447
8710	'SERPINB7'	0.602	0.013332239
3676	'ITGA4'	0.592	9.58E-10
5798	'PTPRN'	0.587	0.007495994
5021	'OXTR'	0.587	0.021368746
51427	'ZNF107'	0.582	0.020366875
57464	'STRIP2'	0.561	9.74E-13
84803	'GPAT3'	0.542	1.69E-06
3778	'KCNMA1'	0.537	1.09E-05
6515	'SLC2A3'	0.533	0.003919858
79899	'PRR5L'	0.532	0.010745974
3655	'ITGA6'	0.528	2.72E-09
214	'ALCAM'	0.513	6.75E-13
2332	'FMR1'	0.509	2.36E-11
11340	'EXOSC8'	0.501	0.013509107
144455	'E2F7'	0.500	0.009001195
50861	'STMN3'	0.487	4.48E-13
2650	'GCNT1'	0.483	0.005521188
116211	'TM4SF19'	0.470	8.35E-16
10846	'PDE10A'	0.463	0.023642401
10418	'SPON1'	0.452	0.018607319
284266	'SIGLEC15'	0.445	4.76E-11
81930	'KIF18A'	0.445	0.005737963
55646	'LYAR'	0.442	3.14E-04
25788	'RAD54B'	0.439	0.003919858

55635	'DEPDC1'	0.439	1.10E-05
10580	'SORBS1'	0.437	0.006143465
89797	'NAV2'	0.435	1.21E-08
10733	'PLK4'	0.435	0.022898554
6525	'SMTN'	0.431	0.022527906
9585	'KIF20B'	0.431	2.75E-04
10381	'TUBB3'	0.430	3.65E-10
6857	'SYT1'	0.430	8.70E-04
1809	'DPYSL3'	0.425	2.57E-08
4810	'NHS'	0.423	0.045024264
4144	'MAT2A'	0.417	1.56E-30
6932	'TCF7'	0.415	6.16E-07
1901	'S1PR1'	0.414	0.001935969
57082	'KNL1'	0.412	0.003112216
55008	'HERC6'	0.411	0.031880145
9212	'AURKB'	0.410	0.02553651
200879	'LIPH'	0.406	0.022921312
5865	'RAB3B'	0.404	3.26E-06
646	'BNC1'	0.398	3.81E-07
9787	'DLGAP5'	0.392	6.97E-04
1428	'CRYM'	0.391	7.33E-04
92949	'ADAMTSL1'	0.390	0.0299167
51765	'STK26'	0.381	0.006169778
22800	'RRAS2'	0.375	9.23E-16
5347	'PLK1'	0.374	2.59E-06
51474	'LIMA1'	0.371	8.02E-12
284021	'MILR1'	0.369	0.017725516
6546	'SLC8A1'	0.368	0.007716555
4085	'MAD2L1'	0.367	3.19E-04
201799	'TMEM154'	0.363	0.010143001
23594	'ORC6'	0.362	0.04988516
83641	'FAM107B'	0.359	4.02E-04
9928	'KIF14'	0.359	1.54E-04
54443	'ANLN'	0.357	3.30E-07
10376	'TUBA1B'	0.356	5.14E-19
5054	'SERPINE1'	0.355	7.98E-06
1062	'CENPE'	0.355	3.70E-04
6607	'SMN2'	0.354	0.001550218
26289	'AK5'	0.354	0.012340452
3489	'IGFBP6'	0.354	0.03936076
10403	'NDC80'	0.353	0.046045247
81553	'CYRIA'	0.353	2.43E-06
5357	'PLS1'	0.352	0.002928808

4603	'MYBL1'	0.351	0.016614468
55355	'HJURP'	0.350	3.61E-05
9319	'TRIP13'	0.349	0.009568502
316	'AOX1'	0.342	5.92E-04
10926	'DBF4'	0.341	0.013188272
55320	'MIS18BP1'	0.340	0.024925357
22974	'TPX2'	0.337	9.70E-12
57650	'CIP2A'	0.336	0.003473445
701	'BUB1B'	0.335	0.001194548
991	'CDC20'	0.335	6.52E-05
55872	'PBK'	0.331	0.017829491
10592	'SMC2'	0.329	8.28E-04
3832	'KIF11'	0.328	6.02E-04
3241	'HPCAL1'	0.326	1.01E-10
891	'CCNB1'	0.326	9.93E-10
118429	'ANTXR2'	0.326	0.017566307
10051	'SMC4'	0.324	1.67E-06
220	'ALDH1A3'	0.323	0.021945526
699	'BUB1'	0.323	4.42E-06
79158	'GNPTAB'	0.322	1.26E-06
4856	'CCN3'	0.322	0.024777572
54504	'CPVL'	0.321	4.12E-04
3833	'KIFC1'	0.319	0.016593663
5627	'PROS1'	0.319	0.013914605
4751	'NEK2'	0.317	0.00911938
4907	'NT5E'	0.317	3.32E-12
1164	'CKS2'	0.316	0.004950327
84675	'TRIM55'	0.316	2.47E-09
55165	'CEP55'	0.315	5.20E-05
1063	'CENPF'	0.314	6.08E-09
23397	'NCAPH'	0.312	0.04405239
222389	'BEND7'	0.312	0.00748514
79054	'TRPM8'	0.311	0.009788846
114569	'MAL2'	0.311	1.86E-04
8061	'FOSL1'	0.310	1.82E-06
90459	'ERI1'	0.308	0.021949525
157313	'CDCA2'	0.308	0.031544533
8091	'HMGA2'	0.305	2.53E-04
6574	'SLC20A1'	0.305	1.01E-10
26586	'CKAP2'	0.305	0.002651622
3161	'HMMR'	0.304	0.003697307
7039	'TGFA'	0.302	1.31E-09
81610	'FAM83D'	0.301	0.010369767

644943	'RASSF10'	0.299	0.010143001
9493	'KIF23'	0.296	0.001069443
4953	'ODC1'	0.293	0.010264975
116441	'TM4SF18'	0.293	2.75E-04
79801	'SHCBP1'	0.292	7.04E-04
7262	'PHLDA2'	0.292	0.020722424
79827	'CLMP'	0.290	3.96E-06
11065	'UBE2C'	0.288	0.011536611
9232	'PTTG1'	0.288	6.08E-04
890	'CCNA2'	0.287	0.024574545
642938	'INSYN2A'	0.286	0.024627189
24137	'KIF4A'	0.281	0.001114158
9700	'ESPL1'	0.280	0.017566307
57458	'TMCC3'	0.280	0.020804401
5329	'PLAUR'	0.279	0.021086127
91607	'SLFN11'	0.278	0.034079902
55711	'FAR2'	0.273	0.022357177
133396	'IL31RA'	0.272	0.003807651
11113	'CIT'	0.272	0.007672289
1894	'ECT2'	0.270	7.48E-05
10383	'TUBB4B'	0.269	9.08E-10
79083	'MLPH'	0.268	0.001443606
4925	'NUCB2'	0.266	0.023026609
1434	'CSE1L'	0.266	1.41E-05
64151	'NCAPG'	0.265	0.006806548
64129	'TINAGL1'	0.265	1.28E-09
113455421	'DERPC'	0.264	0.016263601
84959	'UBASH3B'	0.263	0.002292861
27338	'UBE2S'	0.263	2.02E-05
9982	'FGFBP1'	0.260	4.55E-04
10457	'GPNMB'	0.259	4.62E-04
9768	'PCLAF'	0.258	0.01611448
11004	'KIF2C'	0.258	0.021996908
143888	'POGLUT3'	0.257	0.048604791
3251	'HPRT1'	0.254	0.02553651
28231	'SLCO4A1'	0.254	1.34E-04
84280	'BTBD10'	0.254	0.018111311
9824	'ARHGAP11A'	0.253	0.011579367
9429	'ABCG2'	0.253	0.041683855
55706	'NDC1'	0.253	0.03015198
2171	'FABP5'	0.252	0.00379184
983	'CDK1'	0.251	0.049587978
3925	'STMN1'	0.251	2.34E-05

2966	'GTF2H2'	0.251	0.037362403
23306	'NEMP1'	0.250	0.016428614
558	'AXL'	0.248	2.82E-09
128239	'IQGAP3'	0.248	0.013087474
55075	'UACA'	0.247	2.98E-06
6241	'RRM2'	0.247	0.003919858
29028	'ATAD2'	0.245	0.002622352
7153	'TOP2A'	0.243	1.48E-04
1026	'CDKN1A'	0.242	8.30E-05
6567	'SLC16A2'	0.241	0.033774483
54892	'NCAPG2'	0.241	2.38E-05
10544	'PROCR'	0.240	0.003199139
64167	'ERAP2'	0.238	0.001935969
1719	'DHFR'	0.237	0.010074234
5787	'PTPRB'	0.237	0.019393386
2274	'FHL2'	0.236	3.59E-08
57552	'NCEH1'	0.236	3.74E-05
387882	'C12orf75'	0.236	6.21E-04
1514	'CTSL'	0.236	2.57E-05
84617	'TUBB6'	0.236	2.60E-06
253461	'ZBTB38'	0.236	0.046976264
6949	'TCOF1'	0.235	2.62E-05
23043	'TNIK'	0.234	0.007553564
388610	'TRNP1'	0.233	6.55E-06
3148	'HMGB2'	0.231	0.001935969
8829	'NRP1'	0.231	1.09E-05
55771	'PRR11'	0.231	6.17E-04
3654	'IRAK1'	0.230	5.39E-08
10589	'DRAP1'	0.228	7.65E-04
9055	'PRC1'	0.228	0.003259438
6513	'SLC2A1'	0.228	0.004101052
54801	'HAUS6'	0.228	0.01027938
27347	'STK39'	0.227	0.012695524
50848	'F11R'	0.223	0.003981071
131566	'DCBLD2'	0.222	2.60E-10
2669	'GEM'	0.222	0.028699923
4001	'LMNB1'	0.222	0.048867667
2305	'FOXM1'	0.221	0.009336603
55691	'FRMD4A'	0.221	0.014786848
4502	'MT2A'	0.221	1.02E-05
55704	'CCDC88A'	0.220	0.007304422
7058	'THBS2'	0.220	0.016614468
25939	'SAMHD1'	0.219	0.038212443

3146	'HMGB1'	0.219	1.73E-04
54498	'SMOX'	0.218	0.03936076
1786	'DNMT1'	0.215	0.018607319
8836	'GGH'	0.213	0.004379214
29127	'RACGAP1'	0.212	0.04531585
9603	'NFE2L3'	0.212	0.017566307
10460	'TACC3'	0.212	0.004413022
3838	'KPNA2'	0.212	2.57E-05
54908	'SPDL1'	0.212	0.027539747
3015	'H2AZ1'	0.211	7.37E-04
960	'CD44'	0.211	3.81E-07
29969	'MDFIC'	0.211	0.044571618
1803	'DPP4'	0.209	2.14E-04
9601	'PDIA4'	0.209	1.62E-06
84790	'TUBA1C'	0.209	6.16E-07
2022	'ENG'	0.208	0.001164007
29789	'OLA1'	0.208	0.002024644
3163	'HMOX2'	0.208	0.017552018
140576	'S100A16'	0.208	0.001687408
60559	'SPCS3'	0.208	0.0036793
5708	'PSMD2'	0.207	1.67E-08
4200	'ME2'	0.207	0.034627143
51372	'TMA7'	0.206	0.040585498
51155	'JPT1'	0.205	9.10E-04
259266	'ASPM'	0.203	0.001064476
7184	'HSP90B1'	0.203	6.16E-07
9689	'BZW1'	0.202	2.62E-04
7298	'TYMS'	0.200	0.022264691
203068	'TUBB'	0.199	3.64E-07
3930	'LBR'	0.199	2.52E-04
3875	'KRT18'	0.199	0.002948944
51330	'TNFRSF12A'	0.197	0.006846155
5902	'RANBP1'	0.196	0.025987824
55763	'EXOC1'	0.196	0.046113196
6790	'AURKA'	0.194	0.031323344
970	'CD70'	0.193	0.003845985
84188	'FAR1'	0.192	0.039404721
3159	'HMGA1'	0.191	3.18E-08
332	'BIRC5'	0.191	0.018042363
6240	'RRM1'	0.187	0.020102371
4678	'NASP'	0.187	0.009497521
9748	'SLK'	0.187	0.03178872
4862	'NPAS2'	0.186	0.019234852

286148	'DPY19L4'	0.185	0.038125449
151011	'SEPTIN10'	0.185	0.011578465
7867	'MAPKAPK3'	0.185	0.03792257
928	'CD9'	0.185	0.034627143
262	'AMD1'	0.184	0.039155528
54733	'SLC35F2'	0.184	0.035091602
51119	'SBDS'	0.181	0.010716931
1047	'CLGN'	0.181	0.006899433
7076	'TIMP1'	0.180	0.008909534
29967	'LRP12'	0.180	0.001551754
51203	'NUSAP1'	0.178	0.028817018
5921	'RASA1'	0.177	0.039237848
10509	'SEMA4B'	0.175	0.003919858
9645	'MICAL2'	0.175	0.002948944
3777	'KCNK3'	0.173	0.009589224
483	'ATP1B3'	0.168	0.002768307
7037	'TFRC'	0.168	1.18E-05
3151	'HMGN2'	0.167	0.014570576
7431	'VIM'	0.167	7.82E-07
55103	'RALGPS2'	0.166	0.004519133
30011	'SH3KBP1'	0.165	0.031443742
9918	'NCAPD2'	0.163	0.011674914
25932	'CLIC4'	0.162	0.011578465
9793	'CKAP5'	0.160	0.006800036
9446	'GSTO1'	0.159	0.01177703
10527	'IPO7'	0.158	0.005373244
5899	'RALB'	0.157	0.028609449
23165	'NUP205'	0.157	0.023183949
84823	'LMNB2'	0.155	0.044571618
8204	'NRIP1'	0.154	0.022403141
3910	'LAMA4'	0.152	0.021988007
4071	'TM4SF1'	0.150	0.006073886
3856	'KRT8'	0.150	0.00597335
9747	'TCAF1'	0.150	0.013548856
4176	'MCM7'	0.148	0.03590813
23350	'U2SURP'	0.146	0.020734477
5905	'RANGAP1'	0.145	0.044648966
5757	'PTMA'	0.145	0.002794392
811	'CALR'	0.144	8.60E-04
23204	'ARL6IP1'	0.140	0.027058792
2181	'ACSL3'	0.139	0.020804401
7846	'TUBA1A'	0.138	0.021606928
7052	'TGM2'	0.137	5.41E-04

8140	'SLC7A5'	0.135	0.020203611
1984	'EIF5A'	0.135	0.005328041
5885	'RAD21'	0.135	0.020722424
27122	'DKK3'	0.134	0.024344419
10971	'YWHAQ'	0.132	0.00636149
7468	'NSD2'	0.131	0.033095839
5901	'RAN'	0.128	0.032629636
9411	'ARHGAP29'	0.127	0.017566307
10130	'PDIA6'	0.127	0.020072026
9448	'MAP4K4'	0.124	0.02739527
7170	'TPM3'	0.123	0.003919858
7045	'TGFBI'	0.123	0.011583386
3921	'RPSA'	0.122	0.018859355
1829	'DSG2'	0.122	0.031027127
292	'SLC25A5'	0.121	0.022895391
3309	'HSPA5'	0.121	0.034743968
1917	'EEF1A2'	0.120	0.017566307
7534	'YWHAZ'	0.119	0.00379184
6217	'RPS16'	0.118	0.043851315
191	'AHCY'	0.115	0.03936076
3320	'HSP90AA1'	0.114	0.003780919
1192	'CLIC1'	0.107	0.048604791
3098	'HK1'	0.106	0.046430554
4691	'NCL'	0.104	0.030496395
231	'AKR1B1'	-0.111	0.014345707
1642	'DDB1'	-0.112	0.033095839
65125	'WNK1'	-0.112	0.043388137
3716	'JAK1'	-0.113	0.008010457
10618	'TGOLN2'	-0.113	0.042754921
55959	'SULF2'	-0.117	0.046045247
10075	'HUWE1'	-0.117	0.038024671
4638	'MYLK'	-0.119	0.032783825
57448	'BIRC6'	-0.121	0.044571618
6772	'STAT1'	-0.121	0.003919858
81619	'TSPAN14'	-0.122	0.038387663
8878	'SQSTM1'	-0.125	5.99E-05
2034	'EPAS1'	-0.125	0.010454033
81792	'ADAMTS12'	-0.128	0.030064884
1508	'CTSB'	-0.128	0.028238721
1314	'COPA'	-0.128	0.004640678
307	'ANXA4'	-0.129	0.026535302
3107	'HLA-C'	-0.129	0.015683883
7837	'PXDN'	-0.131	0.047119045

375790	'AGRN'	-0.132	0.023642401
4097	'MAFG'	-0.133	0.029462015
3693	'ITGB5'	-0.133	0.01763788
2539	'G6PD'	-0.133	0.003091257
5660	'PSAP'	-0.135	0.011578465
7086	'TKT'	-0.135	3.80E-04
80243	'PREX2'	-0.136	0.031583414
6721	'SREBF2'	-0.136	0.041325118
64778	'FNDC3B'	-0.136	0.007287915
2760	'GM2A'	-0.137	0.032230799
7077	'TIMP2'	-0.139	0.001902715
55243	'KIRREL1'	-0.139	0.011640816
217	'ALDH2'	-0.140	0.020722424
50717	'DCAF8'	-0.140	0.048684973
79901	'CYBRD1'	-0.140	0.017359414
1785	'DNM2'	-0.140	0.034743968
1282	'COL4A1'	-0.141	0.01914569
9060	'PAPSS2'	-0.141	0.015576318
80727	'TTYH3'	-0.142	0.026035217
5869	'RAB5B'	-0.142	0.037871716
2319	'FLOT2'	-0.143	0.031544533
5236	'PGM1'	-0.143	0.044765502
51429	'SNX9'	-0.143	0.010249637
1267	'CNP'	-0.148	0.046430554
9919	'SEC16A'	-0.149	0.0187972
3106	'HLA-B'	-0.149	0.005591877
80228	'ORAI2'	-0.149	0.014275247
57648	'KIAA1522'	-0.149	0.019313952
3081	'HGD'	-0.150	0.04745001
149603	'RNF187'	-0.150	0.005009701
8566	'PDXK'	-0.150	0.006217991
7461	'CLIP2'	-0.151	0.010249637
23022	'PALLD'	-0.151	0.005145534
10000	'AKT3'	-0.151	0.014162919
23475	'QPRT'	-0.152	2.20E-04
1015	'CDH17'	-0.152	0.047510738
7029	'TFDP2'	-0.152	0.04015253
3685	'ITGAV'	-0.153	2.58E-05
6840	'SVIL'	-0.153	0.01587381
114823	'LENG8'	-0.154	0.026103554
10397	'NDRG1'	-0.154	0.006936642
9019	'MPZL1'	-0.154	7.85E-04
9076	'CLDN1'	-0.156	4.12E-04

57720	'GPR107'	-0.157	0.011817325
23042	'PDXDC1'	-0.158	0.001906107
2247	'FGF2'	-0.158	0.017965691
23185	'LARP4B'	-0.158	0.012503815
6533	'SLC6A6'	-0.159	0.00576805
85456	'TNKS1BP1'	-0.159	0.035667272
3992	'FADS1'	-0.160	4.99E-04
728661	'SLC35E2B'	-0.160	0.011237435
138151	'NACC2'	-0.162	0.042754921
481	'ATP1B1'	-0.162	0.001902715
11261	'CHP1'	-0.163	0.008494052
6624	'FSCN1'	-0.163	7.46E-04
11346	'SYNPO'	-0.163	0.006396406
80114	'BICC1'	-0.164	0.003497252
10160	'FARP1'	-0.164	0.026044659
147166	'TRIM16L'	-0.165	0.013308638
3315	'HSPB1'	-0.166	4.89E-04
3949	'LDLR'	-0.166	0.006289358
81502	'HM13'	-0.167	0.004519194
220002	'CYB561A3'	-0.167	0.032225996
22839	'DLGAP4'	-0.167	0.038208339
7057	'THBS1'	-0.168	0.020203611
151887	'CCDC80'	-0.168	0.013569831
138050	'HGSNAT'	-0.168	0.026803027
55108	'BSDC1'	-0.169	0.037271199
3105	'HLA-A'	-0.169	1.29E-04
4891	'SLC11A2'	-0.170	0.021454473
2222	'FDFT1'	-0.170	0.002775399
1595	'CYP51A1'	-0.171	0.001104005
3725	'JUN'	-0.171	0.028817018
283149	'BCL9L'	-0.171	0.044736565
1523	'CUX1'	-0.171	0.004063774
10318	'TNIP1'	-0.171	1.15E-04
79776	'ZFHX4'	-0.171	0.045507954
5332	'PLCB4'	-0.171	4.36E-04
26020	'LRP10'	-0.173	0.005240124
23743	'BHMT2'	-0.173	0.001591571
1186	'CLCN7'	-0.173	0.00670715
6713	'SQLE'	-0.174	0.00766429
48	'ACO1'	-0.174	4.68E-04
1476	'CSTB'	-0.175	0.005240124
4067	'LYN'	-0.175	0.044927838
2355	'FOSL2'	-0.175	1.43E-04

1605	'DAG1'	-0.175	0.003981071
57493	'HEG1'	-0.175	0.002107252
64359	'NXN'	-0.176	0.0049533
8804	'CREG1'	-0.176	3.46E-04
6888	'TALDO1'	-0.176	2.01E-04
25999	'CLIP3'	-0.176	0.037765674
23295	'MGRN1'	-0.178	0.022213506
112	'ADCY6'	-0.179	0.029288867
84168	'ANTXR1'	-0.179	0.015576318
79650	'USB1'	-0.179	0.005246215
9620	'CELSR1'	-0.179	0.023909545
23275	'POFUT2'	-0.179	0.045994586
8321	'FZD1'	-0.179	0.045594729
83692	'CD99L2'	-0.180	0.020309367
20	'ABCA2'	-0.181	0.01611448
3491	'CCN1'	-0.181	2.71E-04
9907	'AP5Z1'	-0.181	0.039124086
9445	'ITM2B'	-0.182	1.77E-07
54868	'TMEM104'	-0.182	0.045994586
10628	'TXNIP'	-0.183	0.044927838
1284	'COL4A2'	-0.184	2.97E-04
2632	'GBE1'	-0.184	0.004299853
10150	'MBNL2'	-0.184	0.046795125
7168	'TPM1'	-0.184	1.56E-06
5833	'PCYT2'	-0.184	0.020203611
2720	'GLB1'	-0.185	0.003981071
1759	'DNM1'	-0.185	0.001103678
100133941	'CD24'	-0.186	0.002622352
4905	'NSF'	-0.187	0.021788743
55238	'SLC38A7'	-0.187	0.048604791
1200	'TPP1'	-0.187	3.92E-04
4257	'MGST1'	-0.187	2.95E-04
10608	'MXD4'	-0.187	0.043297034
81603	'TRIM8'	-0.190	0.003053334
54472	'TOLLIP'	-0.190	0.025653036
8555	'CDC14B'	-0.190	0.026035217
3340	'NDST1'	-0.190	2.62E-04
4781	'NFIB'	-0.190	0.005931438
23286	'WWC1'	-0.190	2.72E-04
6383	'SDC2'	-0.191	0.013569831
3064	'HTT'	-0.191	8.76E-04
120	'ADD3'	-0.191	2.73E-05
2717	'GLA'	-0.191	0.019854785

84255	'SLC37A3'	-0.192	0.006846155
78996	'CYREN'	-0.193	7.37E-04
7764	'ZNF217'	-0.194	5.99E-05
53373	'TPCN1'	-0.194	7.12E-04
2896	'GRN'	-0.195	2.12E-04
55819	'RNF130'	-0.195	0.045848265
11337	'GABARAP'	-0.195	0.003259438
3490	'IGFBP7'	-0.196	6.65E-07
1845	'DUSP3'	-0.196	0.004299853
23013	'SPEN'	-0.197	0.002345757
11282	'MGAT4B'	-0.198	1.13E-06
1808	'DPYSL2'	-0.198	0.001516037
3304	'HSPA1B'	-0.199	0.006177594
11245	'GPR176'	-0.200	0.02410747
65985	'AACS'	-0.200	0.034299473
8828	'NRP2'	-0.200	0.002528811
2192	'FBLN1'	-0.201	0.032021394
9249	'DHRS3'	-0.201	0.048684973
23158	'TBC1D9'	-0.202	0.003971228
221935	'SDK1'	-0.202	0.030302924
23654	'PLXNB2'	-0.203	3.87E-05
9820	'CUL7'	-0.203	0.036672691
54461	'FBXW5'	-0.203	0.003005078
2548	'GAA'	-0.203	0.008010457
5797	'PTPRM'	-0.203	2.43E-05
10327	'AKR1A1'	-0.204	0.003988218
3157	'HMGCS1'	-0.205	0.007287915
51186	'TCEAL9'	-0.205	0.002623688
8897	'MTMR3'	-0.207	0.047038353
729993	'SHISA9'	-0.207	0.001516037
125058	'TBC1D16'	-0.208	0.001413623
60681	'FKBP10'	-0.209	4.44E-06
51232	'CRIM1'	-0.211	1.48E-06
54893	'MTMR10'	-0.211	0.047092102
3303	'HSPA1A'	-0.214	4.83E-04
55353	'LAPTM4B'	-0.214	1.11E-08
6774	'STAT3'	-0.215	0.00435948
9380	'GRHPR'	-0.215	0.002736405
8814	'CDKL1'	-0.216	0.047211649
9693	'RAPGEF2'	-0.217	0.002536327
6497	'SKI'	-0.218	0.026990445
140809	'SRXN1'	-0.218	1.90E-05
7296	'TXNRD1'	-0.218	1.53E-09

91373	'UAP1L1'	-0.219	0.016341371
30850	'CDR2L'	-0.221	8.10E-04
1717	'DHCR7'	-0.222	2.12E-04
23710	'GABARAPL1'	-0.222	0.001584559
10221	'TRIB1'	-0.222	0.004101052
55608	'ANKRD10'	-0.223	0.002662201
780	'DDR1'	-0.223	0.001112231
26471	'NUPR1'	-0.224	8.62E-04
10217	'CTDSPL'	-0.224	0.00212353
3709	'ITPR2'	-0.225	3.46E-04
3624	'INHBA'	-0.226	2.05E-04
3569	'IL6'	-0.227	0.003554761
1612	'DAPK1'	-0.229	0.001581645
9536	'PTGES'	-0.229	0.028061848
9516	'LITAF'	-0.230	8.99E-10
114907	'FBXO32'	-0.230	2.73E-04
6309	'SC5D'	-0.231	3.23E-05
5226	'PGD'	-0.231	4.10E-09
4779	'NFE2L1'	-0.231	4.27E-06
7127	'TNFAIP2'	-0.232	3.44E-11
649	'BMP1'	-0.233	0.001443606
146802	'SLC47A2'	-0.233	0.043297034
4035	'LRP1'	-0.233	5.77E-06
343702	'XKR7'	-0.235	0.035957436
347902	'AMIGO2'	-0.236	5.32E-08
4597	'MVD'	-0.236	4.30E-04
84662	'GLIS2'	-0.236	0.0036793
5834	'PYGB'	-0.237	3.97E-09
54921	'CHTF8'	-0.237	9.10E-04
9902	'MRC2'	-0.237	2.03E-07
7424	'VEGFC'	-0.238	0.005057042
9194	'SLC16A7'	-0.238	0.009788846
23328	'SASH1'	-0.238	0.025822259
10625	'IVNS1ABP'	-0.239	2.56E-05
10584	'COLEC10'	-0.239	2.72E-08
2131	'EXT1'	-0.239	8.89E-08
54578	'UGT1A6'	-0.240	1.48E-06
84886	'C1orf198'	-0.242	0.009640035
411	'ARSB'	-0.242	0.026044659
3480	'IGF1R'	-0.243	0.001936376
4897	'NRCAM'	-0.243	1.31E-07
9971	'NR1H4'	-0.244	0.006206963
7084	'TK2'	-0.245	0.0036793

4363	'ABCC1'	-0.247	5.72E-07
3459	'IFNGR1'	-0.248	0.006707445
718	'C3'	-0.248	6.65E-07
847	'CAT'	-0.248	3.40E-06
7035	'TFPI'	-0.248	4.71E-09
2224	'FDPS'	-0.249	7.14E-06
3696	'ITGB8'	-0.251	8.76E-04
9563	'H6PD'	-0.251	4.70E-04
3257	'HPS1'	-0.253	0.036585433
51421	'AMOTL2'	-0.254	6.71E-07
2335	'FN1'	-0.254	1.76E-08
6817	'SULT1A1'	-0.261	0.003199139
415	'ARSL'	-0.261	6.67E-04
3694	'ITGB6'	-0.262	0.02110165
54972	'TMEM132A'	-0.263	3.68E-06
10602	'CDC42EP3'	-0.264	0.017874046
9686	'VGLL4'	-0.264	2.22E-04
133746	'JMY'	-0.266	0.018408304
10417	'SPON2'	-0.266	6.16E-07
9435	'CHST2'	-0.266	0.023620648
7439	'BEST1'	-0.268	0.010816553
3913	'LAMB2'	-0.268	2.53E-06
2634	'GBP2'	-0.270	0.016374051
22795	'NID2'	-0.273	4.46E-04
56977	'STOX2'	-0.274	0.005206847
2182	'ACSL4'	-0.274	1.56E-11
1051	'CEBPB'	-0.275	8.46E-09
55902	'ACSS2'	-0.275	4.78E-08
4047	'LSS'	-0.278	1.44E-09
81788	'NUAK2'	-0.286	1.17E-04
144132	'DNHD1'	-0.286	0.020547921
55188	'RIC8B'	-0.286	0.021924433
169792	'GLIS3'	-0.288	8.60E-04
10891	'PPARGC1A'	-0.288	0.004645388
65249	'ZSWIM4'	-0.288	0.037076623
64759	'TNS3'	-0.290	3.72E-10
1307	'COL16A1'	-0.290	5.38E-08
57326	'PBXIP1'	-0.291	2.91E-07
1195	'CLK1'	-0.291	0.024777572
5243	'ABCB1'	-0.291	0.039470569
84909	'AOPEP'	-0.293	0.009336603
6764	'DENND2B'	-0.293	6.31E-06
216	'ALDH1A1'	-0.293	5.27E-07

716	'C1S'	-0.296	7.67E-10
5909	'RAP1GAP'	-0.297	0.019393386
3727	'JUND'	-0.297	0.004971018
23117	'NPIPB3'	-0.298	0.012209902
10398	'MYL9'	-0.298	0.001104149
114897	'C1QTNF1'	-0.300	0.001089621
3691	'ITGB4'	-0.302	0.02677963
9056	'SLC7A7'	-0.302	0.002947275
8714	'ABCC3'	-0.302	2.64E-09
4792	'NFKBIA'	-0.302	1.08E-04
1435	'CSF1'	-0.303	3.32E-11
8500	'PPFIA1'	-0.305	8.22E-12
1891	'ECH1'	-0.305	2.02E-07
1294	'COL7A1'	-0.311	0.011205802
80763	'SPX'	-0.312	1.69E-05
3667	'IRS1'	-0.313	3.59E-04
2185	'PTK2B'	-0.315	1.22E-05
715	'C1R'	-0.315	2.21E-07
29057	'FAM156A'	-0.316	3.54E-04
9900	'SV2A'	-0.316	5.26E-05
63874	'ABHD4'	-0.316	7.27E-05
6659	'SOX4'	-0.318	0.00677104
55753	'OGDHL'	-0.318	0.001936376
22949	'PTGR1'	-0.318	1.42E-13
10014	'HDAC5'	-0.318	0.007884097
5140	'PDE3B'	-0.319	0.015087243
84898	'PLXDC2'	-0.322	1.08E-05
1362	'CPD'	-0.324	9.23E-16
6288	'SAA1'	-0.328	4.24E-10
460	'ASTN1'	-0.330	0.024344419
5754	'PTK7'	-0.331	0.023414721
6303	'SAT1'	-0.333	1.55E-08
89932	'PAPLN'	-0.337	7.61E-05
81794	'ADAMTS10'	-0.338	0.014243175
92017	'SNX29'	-0.338	7.56E-05
6604	'SMARCD3'	-0.341	0.040585498
80014	'WWC2'	-0.342	2.12E-12
11213	'IRAK3'	-0.342	7.69E-04
117583	'PARD3B'	-0.345	0.003058356
10158	'PDZK1IP1'	-0.348	2.16E-19
4038	'LRP4'	-0.349	7.19E-11
57016	'AKR1B10'	-0.349	4.54E-16
57210	'SLC45A4'	-0.350	0.024925357

83937	'RASSF4'	-0.350	2.03E-06
5649	'RELN'	-0.351	1.52E-08
6608	'SMO'	-0.351	6.65E-07
10135	'NAMPT'	-0.352	2.66E-14
2052	'EPHX1'	-0.355	4.17E-16
4133	'MAP2'	-0.356	9.74E-04
340348	'TSPAN33'	-0.359	2.69E-10
1410	'CRYAB'	-0.360	1.85E-05
63027	'SLC22A23'	-0.361	0.032750821
130367	'SGPP2'	-0.364	2.02E-07
1827	'RCAN1'	-0.368	0.001806928
8644	'AKR1C3'	-0.369	9.85E-14
343578	'ARHGAP40'	-0.371	1.28E-09
79605	'PGBD5'	-0.375	1.56E-11
1952	'CELSR2'	-0.377	0.006169778
440712	'RHEX'	-0.377	0.00265109
64856	'VWA1'	-0.380	0.021072568
3207	'HOXA11'	-0.382	0.003443085
57514	'ARHGAP31'	-0.384	3.59E-08
9242	'MSC'	-0.384	2.42E-07
222962	'SLC29A4'	-0.384	0.00131667
114990	'VASN'	-0.386	0.022742807
9415	'FADS2'	-0.388	2.08E-12
596	'BCL2'	-0.389	0.023189793
6581	'SLC22A3'	-0.394	5.90E-04
26253	'CLEC4E'	-0.395	1.42E-10
85004	'RERG'	-0.396	0.021788743
6446	'SGK1'	-0.398	0.002074487
10893	'MMP24'	-0.398	4.93E-05
57722	'IGDCC4'	-0.400	0.001180684
3728	'JUP'	-0.400	3.03E-14
144165	'PRICKLE1'	-0.401	0.008580418
619279	'ZNF704'	-0.402	0.001962577
1999	'ELF3'	-0.412	2.77E-10
26232	'FBXO2'	-0.416	0.028722365
25987	'TSKU'	-0.416	2.71E-13
56937	'PMEPA1'	-0.418	1.88E-13
5266	'PI3'	-0.419	0.02739527
4881	'NPR1'	-0.422	1.16E-07
645369	'TMEM200C'	-0.423	1.43E-04
9922	'IQSEC1'	-0.427	3.59E-05
80144	'FRAS1'	-0.431	8.09E-06
441282	'AKR1B15'	-0.436	0.043297034

113878	'DTX2'	-0.437	2.59E-10
56099	'PCDHGB7'	-0.438	0.026044659
115811	'IQCD'	-0.441	1.31E-07
135	'ADORA2A'	-0.442	0.011205802
6648	'SOD2'	-0.450	2.46E-31
8876	'VNN1'	-0.457	0.03962465
79095	'BBLN'	-0.462	8.23E-09
11320	'MGAT4A'	-0.463	2.37E-04
80149	'ZC3H12A'	-0.465	6.65E-12
6337	'SCNN1A'	-0.471	8.42E-13
81832	'NETO1'	-0.472	5.37E-04
3773	'KCNJ16'	-0.472	3.78E-23
6376	'CX3CL1'	-0.473	1.04E-20
115362	'GBP5'	-0.474	0.030064884
8660	'IRS2'	-0.482	3.39E-04
81543	'LRRC3'	-0.492	0.002177967
629	'CFB'	-0.496	1.23E-05
1645	'AKR1C1'	-0.497	1.81E-30
11309	'SLCO2B1'	-0.511	3.37E-22
6374	'CXCL5'	-0.515	1.31E-09
6289	'SAA2'	-0.516	1.08E-32
4137	'MAPT'	-0.518	0.001410737
5730	'PTGDS'	-0.531	2.09E-08
115701	'ALPK2'	-0.533	1.80E-12
64218	'SEMA4A'	-0.537	0.03319816
23554	'TSPAN12'	-0.550	4.91E-11
4324	'MMP15'	-0.551	2.69E-07
23220	'DTX4'	-0.557	0.0187972
2878	'GPX3'	-0.561	4.51E-16
91683	'SYT12'	-0.562	7.72E-11
6236	'RRAD'	-0.586	0.022527906
4316	'MMP7'	-0.590	1.22E-21
366	'AQP9'	-0.595	3.33E-07
728	'C5AR1'	-0.597	0.029287802
93082	'NEURL3'	-0.601	4.46E-04
55286	'C4orf19'	-0.613	2.15E-07
3576	'CXCL8'	-0.627	2.53E-06
55567	'DNAH3'	-0.628	0.009336603
146433	'IL34'	-0.630	3.23E-05
255488	'RNF144B'	-0.633	1.43E-10
9388	'LIPG'	-0.649	0.002561998
3934	'LCN2'	-0.654	5.28E-04
57795	'BRINP2'	-0.661	2.33E-05

64084	'CLSTN2'	-0.663	5.31E-39
56971	'CEACAM19'	-0.669	0.022219952
10129	'FRY'	-0.673	3.90E-05
7373	'COL14A1'	-0.674	3.76E-45
1850	'DUSP8'	-0.695	0.036557543
1014	'CDH16'	-0.696	1.91E-04
312	'ANXA13'	-0.708	8.60E-04
55790	'CSGALNACT1'	-0.731	1.78E-04
26298	'EHF'	-0.732	0.001591571
90427	'BMF'	-0.741	0.001004118
1646	'AKR1C2'	-0.786	9.34E-35
222865	'TMEM130'	-0.797	1.03E-16
84419	'C15orf48'	-0.806	9.61E-21
100528062	'ARMCX5-GPRASP2'	-0.838	0.020203611
124872	'B4GALNT2'	-0.847	3.67E-15
4739	'NEDD9'	-0.897	0.006196305
79931	'TNIP3'	-0.912	0.004617446
1672	'DEFB1'	-0.991	0.022345409
2919	'CXCL1'	-1.126	2.26E-25
25960	'ADGRA2'	-1.167	8.62E-07
7226	'TRPM2'	-1.264	5.16E-04
6372	'CXCL6'	-1.322	8.32E-20
51450	'PRRX2'	-1.408	0.032460631
1116	'CHI3L1'	-1.626	0.001692937
79853	'TM4SF20'	-2.011	0.02182378
100631383	'FAM47E-STBD1'	-6.910	5.72E-06
