# Supplementary material for: FDX1 overexpression inhibits the growth and metastasis of clear cell renal cell carcinoma by upregulating FMR1 expression
Source: Cell Death Discov. 2025 Mar 21;11:115. doi: 10.1038/s41420-025-02380-5 (PMC11928736; doi:10.1038/s41420-025-02380-5)
Supplement: Supplementary file 4 — Full and uncropped western blots [file 41420_2025_2380_MOESM4_ESM.pdf]

Full and uncropped western blots

Fig.2D

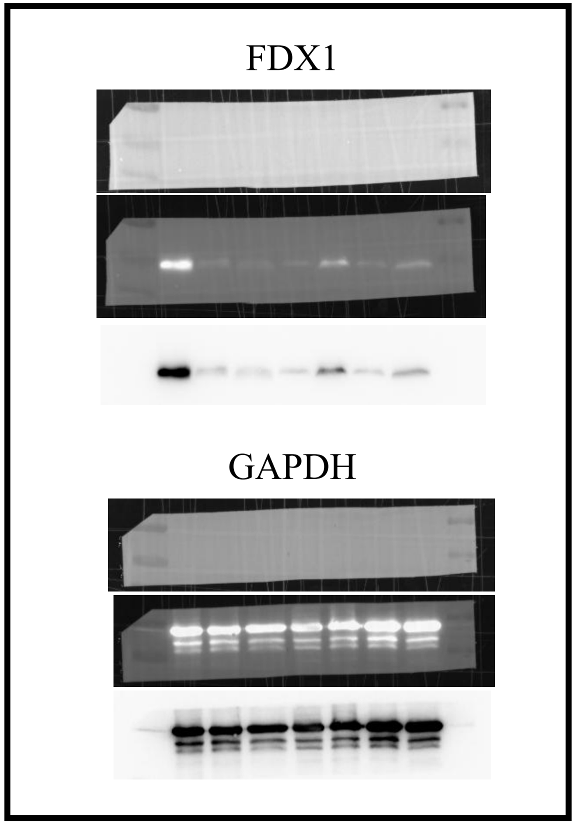

Fig.3A

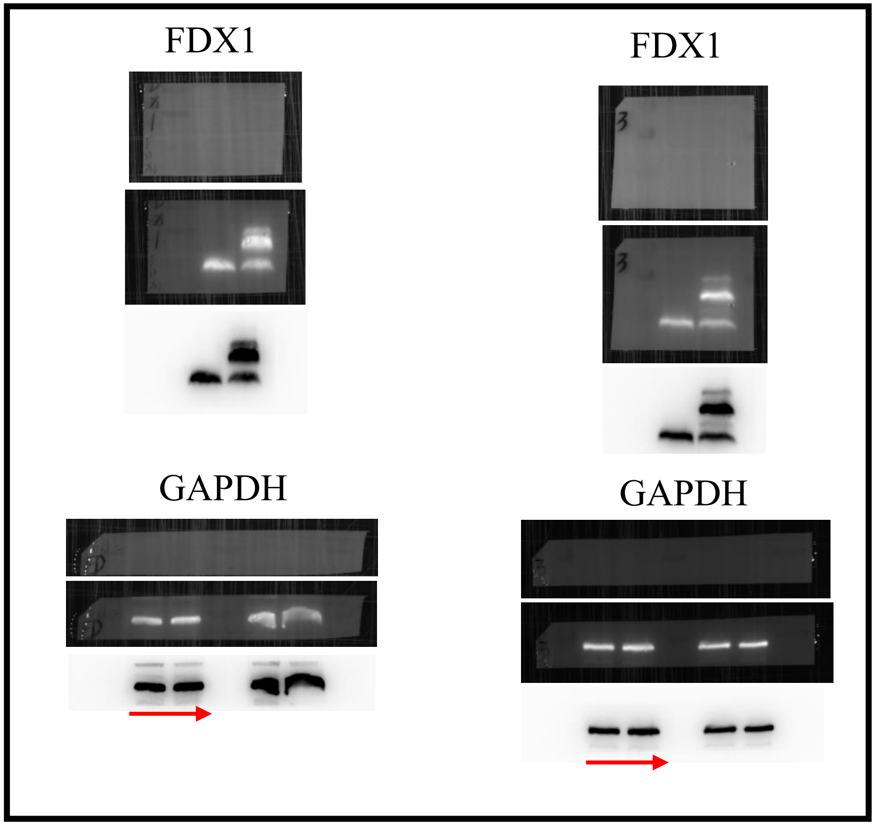

Fig.4A

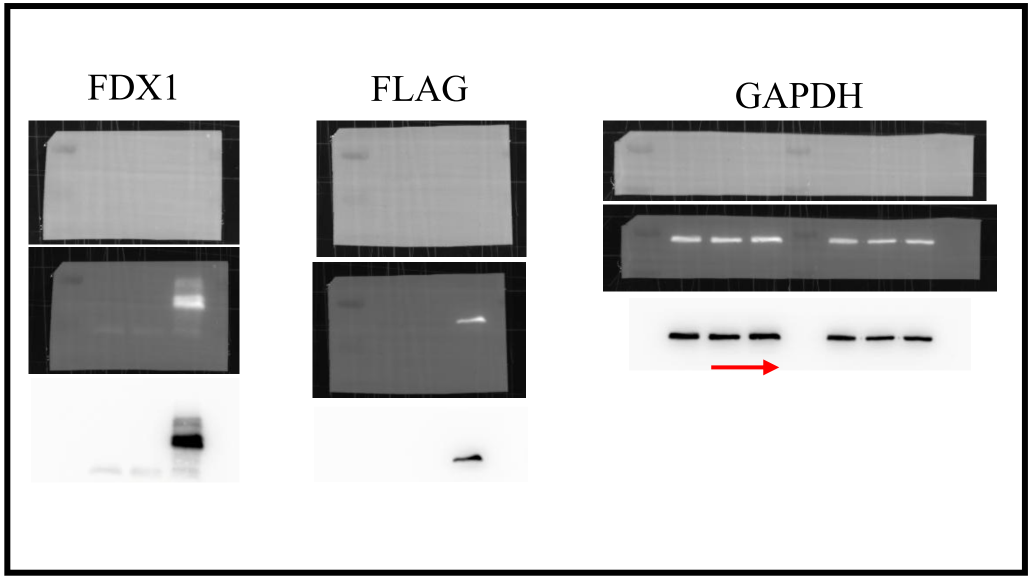

Fig.4B

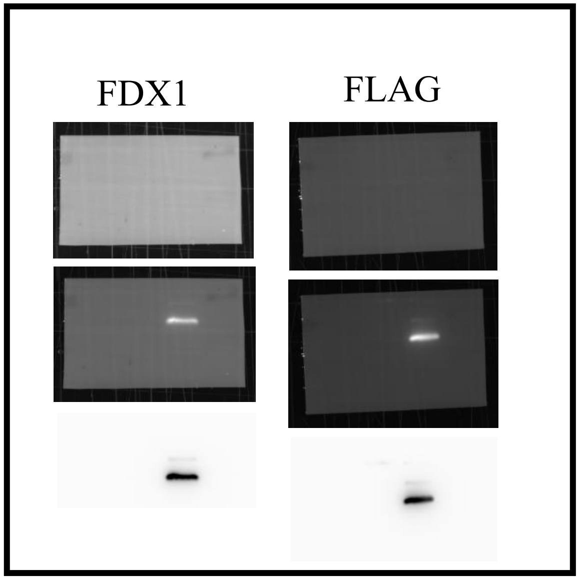

Fig.4F

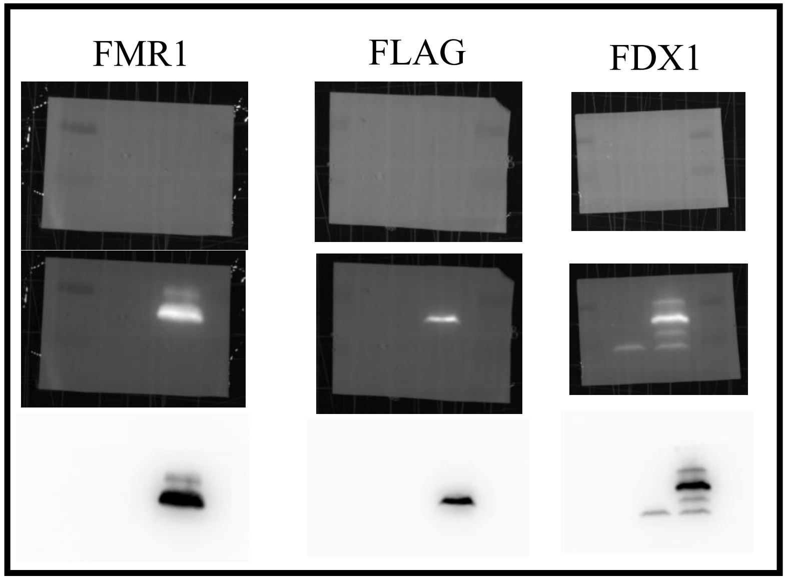

Fig.4G

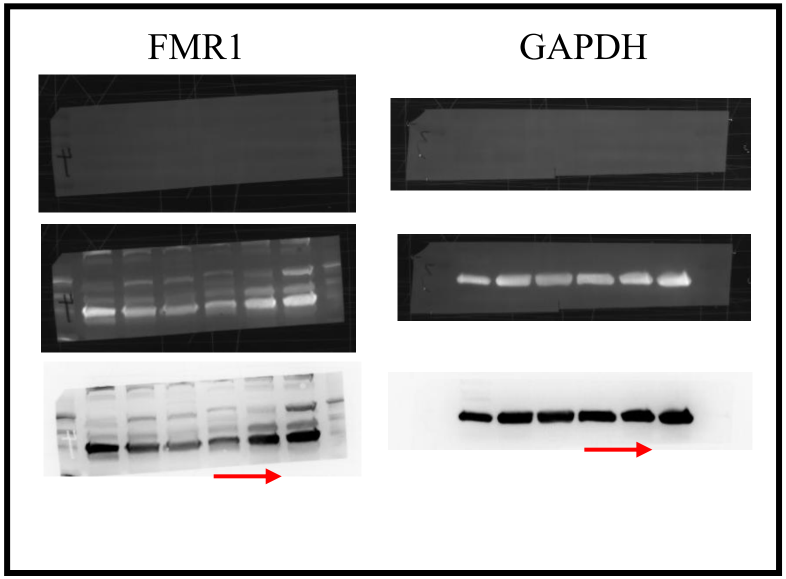

Fig.6A-Input

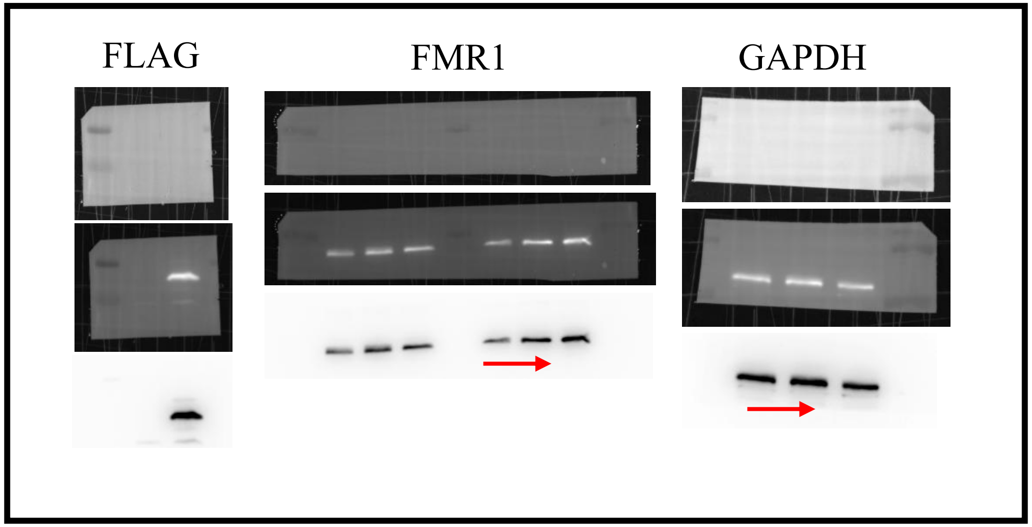

Fig.6A-IP

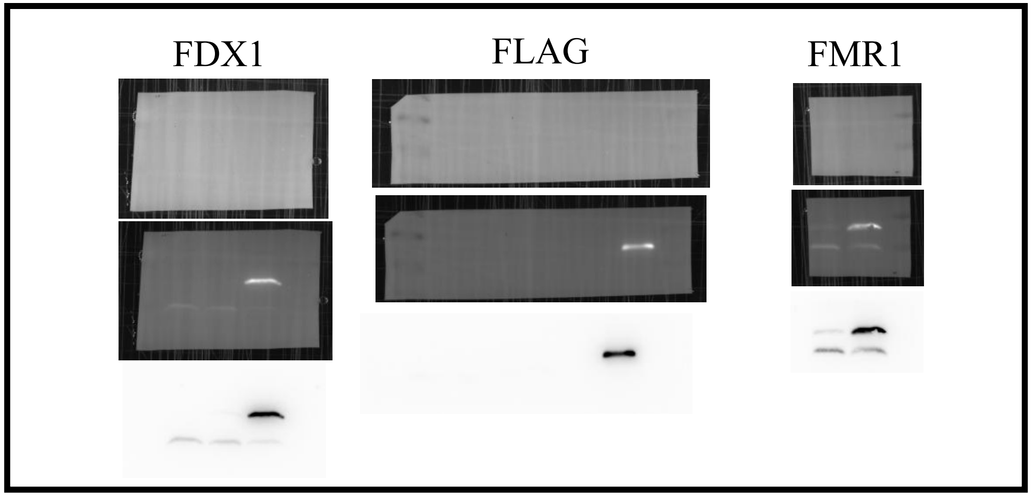

Fig.8A

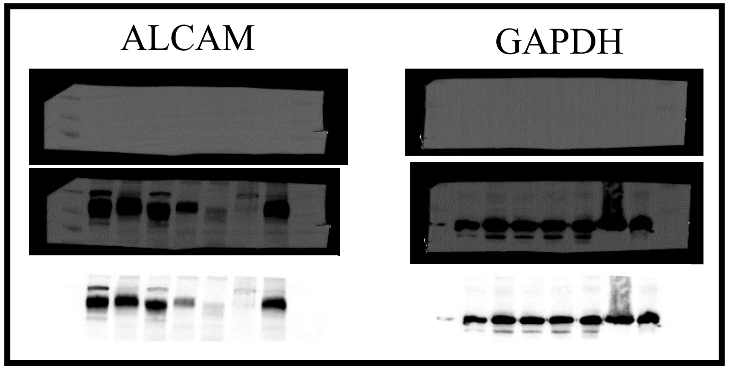

Fig.8B

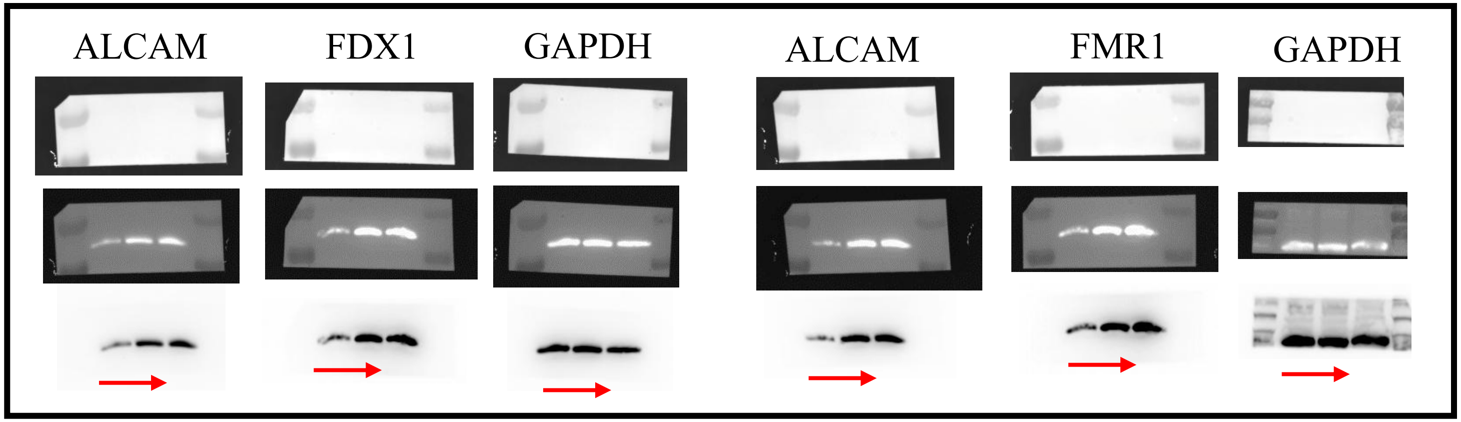

Fig.8C

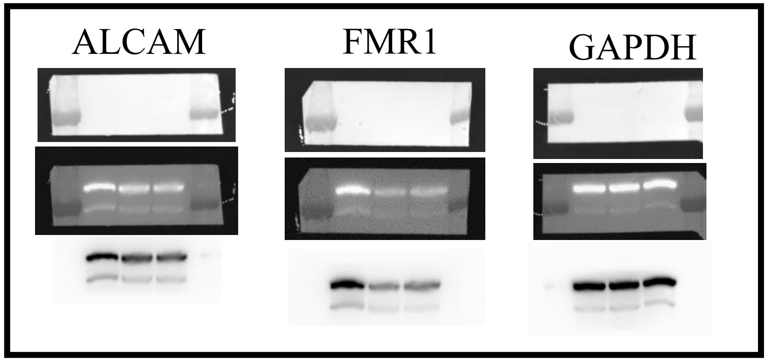

Fig.8D

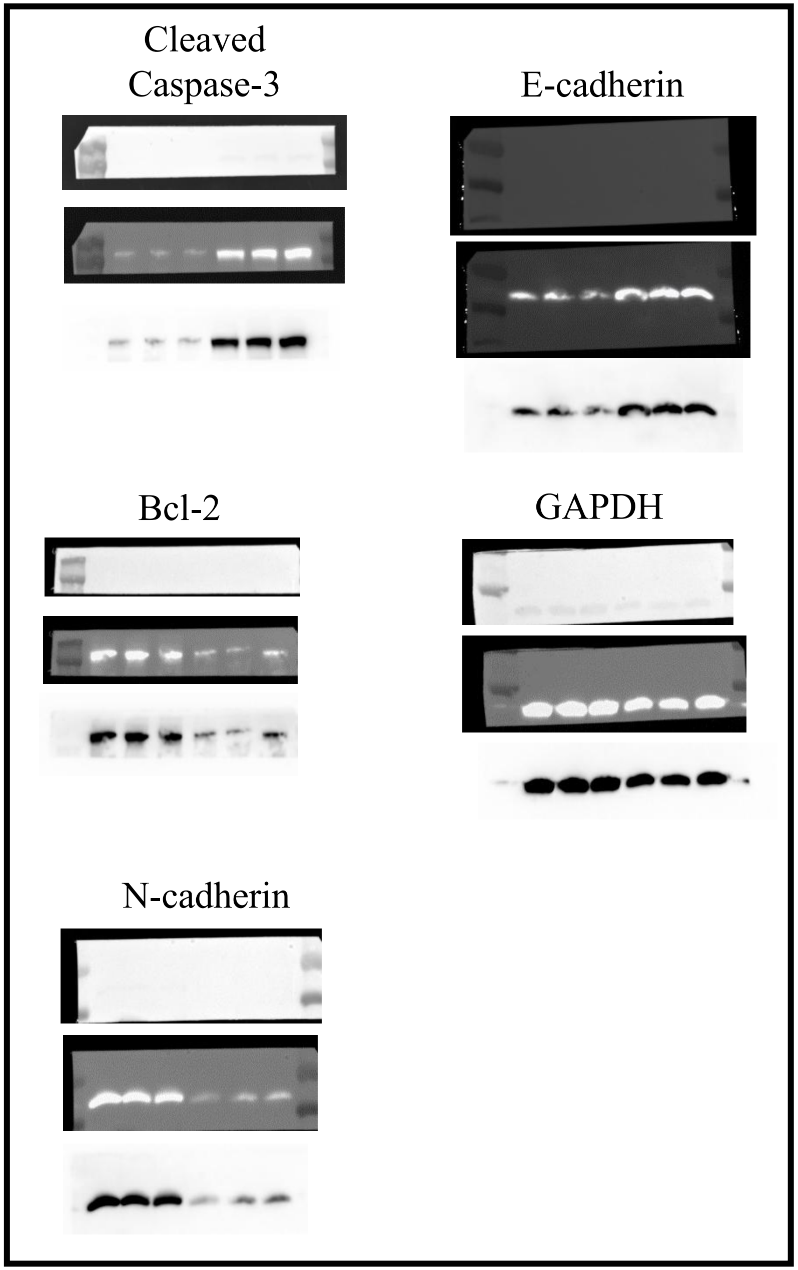
Cleaved Caspase-3
E-cadherin
Bcl-2
GAPDH
N-cadherin

Fig.8E

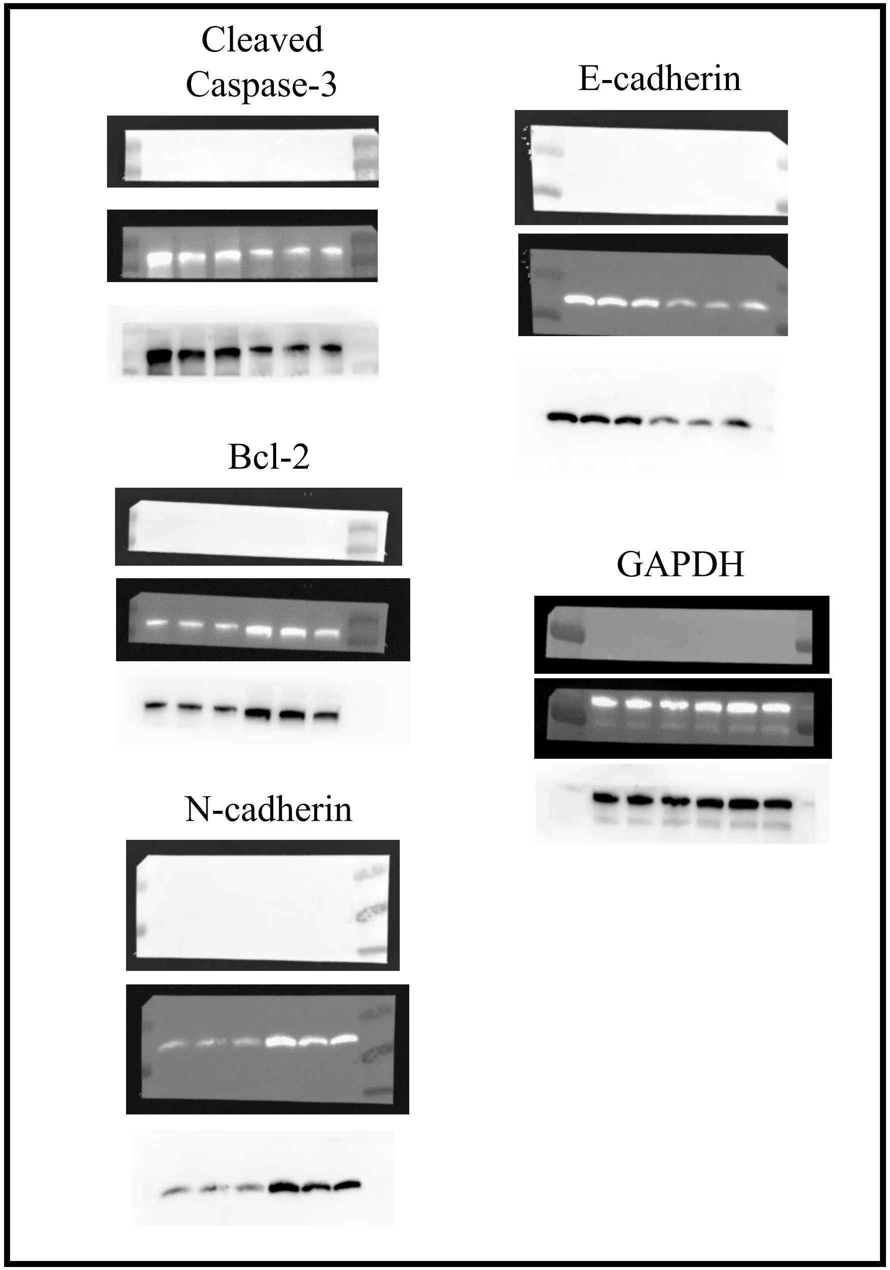
Cleaved Caspase-3
E-cadherin
Bcl-2
GAPDH
N-cadherin
